# Suppression of respiratory growth defect of mitochondrial phosphatidylserine decarboxylase deficient mutant by overproduction of Sfh1, a Sec14 homolog, in yeast

**DOI:** 10.1371/journal.pone.0215009

**Published:** 2019-04-08

**Authors:** Aya Mizuike, Shingo Kobayashi, Takashi Rikukawa, Akinori Ohta, Hiroyuki Horiuchi, Ryouichi Fukuda

**Affiliations:** 1 Department of Biotechnology, The University of Tokyo, Bunkyo-ku, Tokyo, Japan; 2 Department of Biological Chemistry, College of Bioscience and Biotechnology, Chubu University, Kasugai, Aichi, Japan; 3 Collaborative Research Institute for Innovative Microbiology, The University of Tokyo, Bunkyo-ku, Tokyo, Japan; CNR, ITALY

## Abstract

Interorganelle phospholipid transfer is critical for eukaryotic membrane biogenesis. In the yeast *Saccharomyces cerevisiae*, phosphatidylserine (PS) synthesized by PS synthase, Pss1, in the endoplasmic reticulum (ER) is decarboxylated to phosphatidylethanolamine (PE) by PS decarboxylase, Psd1, in the ER and mitochondria or by Psd2 in the endosome, Golgi, and/or vacuole, but the mechanism of interorganelle PS transport remains to be elucidated. Here we report that Sfh1, a member of Sec14 family proteins of *S*. *cerevisiae*, possesses the ability to enhance PE production by Psd2. Overexpression of *SFH1* in the strain defective in Psd1 restored its growth on non-fermentable carbon sources and increased the intracellular and mitochondrial PE levels. Sfh1 was found to bind various phospholipids, including PS, *in vivo*. Bacterially expressed and purified Sfh1 was suggested to have the ability to transport fluorescently labeled PS between liposomes by fluorescence dequenching assay *in vitro*. Biochemical subcellular fractionation suggested that a fraction of Sfh1 localizes to the endosome, Golgi, and/or vacuole. We propose a model that Sfh1 promotes PE production by Psd2 by transferring phospholipids between the ER and endosome.

## Introduction

**Fig 1:**
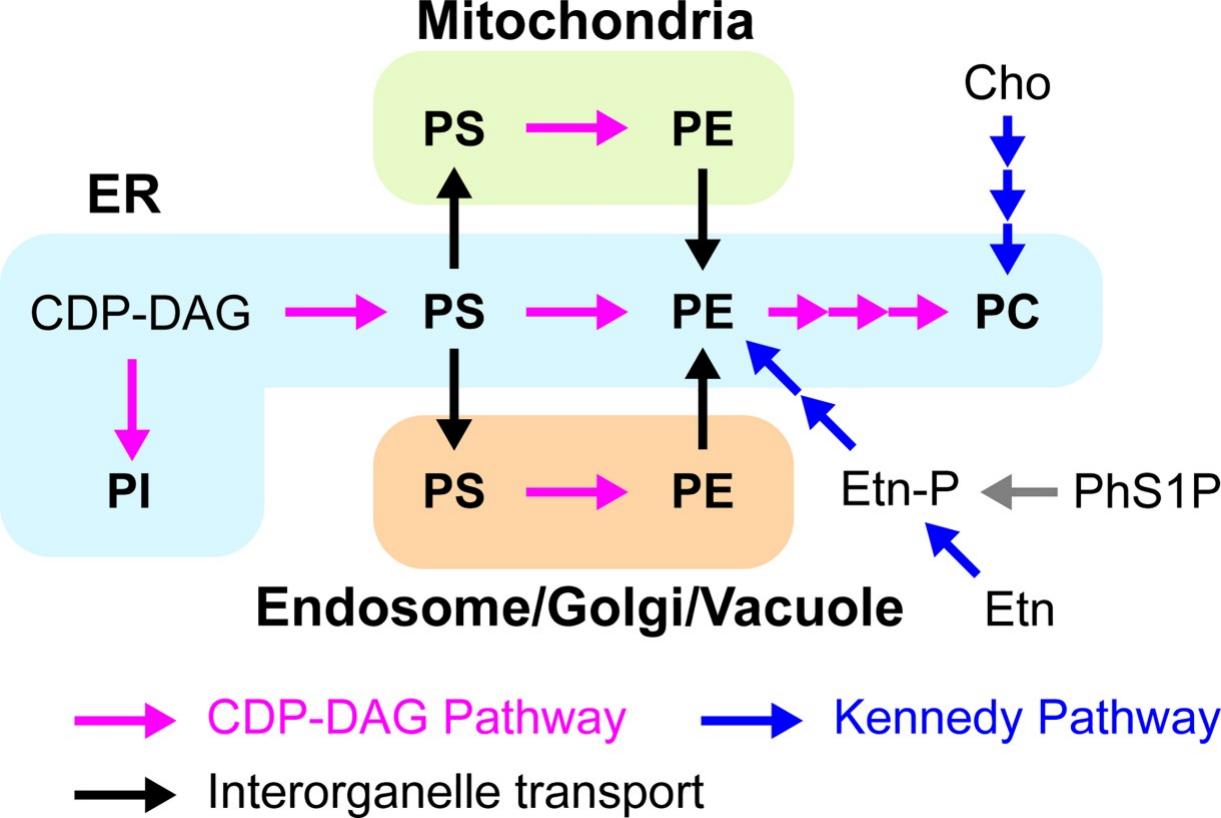
Synthetic pathways of major phospholipids in *S*. *cerevisiae*. Magenta and blue arrows represent reactions of the CDP-DAG pathway and the Kennedy pathway, respectively. Black arrows represent interorganellar phospholipid transport.

In eukaryotic cells, enzymes involved in phospholipid biosynthesis localize to defined organelles, and therefore interorganelle lipid transport is critical for the biogenesis of membranes. Organellar membranes exhibit distinct lipid compositions, which influence the structures and functions of those membranes [1, 2]. The unequal distribution of phospholipids among the organelles and the plasma membrane are generated and maintained by the local synthesis and metabolism of each phospholipid species [3, 4] and the directional transport of phospholipids between membranes. Therefore, interorganellar lipid transport plays an important role in the maintenance of membrane homeostasis. In the budding yeast *Saccharomyces cerevisiae*, interorganelle lipid transport is involved in phospholipid synthesis by the CDP-diacylglycerol (CDP-DAG) pathway (Fig 1). In this pathway, phosphatidylserine (PS) is synthesized from CDP-DAG and serine by PS synthase, Pss1, in the endoplasmic reticulum (ER) membrane [5, 6]. PS is then decarboxylated to phosphatidylethanolamine (PE) by PS decarboxylase, Psd1, in the mitochondria or the ER, or by Psd2 in the endosome, Golgi, and/or vacuole [7–11]. A portion of PE is methylated to phosphatidylcholine (PC) by the PE methyltransferases, Pem1 and Pem2 [12]. PE and PC are also synthesized through the Kennedy pathway from ethanolamine (Etn), phosphoethanolamine, choline (Cho), or phosphocholine in the ER and the Golgi apparatus [13–17].

Membrane lipids are transferred between organelles in vesicular traffic-dependent and -independent manners [18]. Membrane contact sites (MCSs) and lipid transfer proteins (LTPs), which are not mutually exclusive, have been proposed to be involved in non-vesicular lipid transfer [19–21]. Mitochondria are regarded as vesicular traffic-independent organelles, and hence non-vesicular lipid transfer is critical for the biogenesis and maintenance of mitochondrial membranes. Indeed, studies have shown that the mitochondria form MCSs with various organelles. In *S*. *cerevisiae*, ER-mitochondria encounter structure (ERMES) and ER membrane protein complex (EMC) form contacts between the mitochondria and the ER [22, 23]. A contact site named vacuole and mitochondria patch (vCLAMP), marked by Vps39 and Ypt7, is formed between the mitochondria and vacuole [24, 25]. In addition, Vps13 tethers the mitochondria to the endosome and vacuole [26, 27]. Recently, the mammalian homologs of Vps13 were reported to localize at contacts between the ER and mitochondria, late endosome/lysosomes, or lipid droplet, and possess the ability to transfer phospholipids *in vitro* [28]. It was also shown that mitochondria and peroxisome form contact (PerMit) by Fzo1 and Pex34 [29]. These contacts are considered to be involved in phospholipid trafficking, yet their precise functions and regulatory mechanisms remain to be elucidated.

Although the fundamental role of LTPs in non-vesicular lipid transfer has also remained elusive, it has been proposed that rectification of the direction of lipid flux may be the role of LTPs [30]. Sec14, which possesses the ability to transfer phosphatidylinositol (PI) and PC between membranes [PI transfer protein (PITP) and PC transfer protein (PCTP) activities, respectively] *in vitro* [31], and Sec14-family proteins are potential candidates of LTPs that mediate interorganellar phospholipid transport. The Sec14 family of proteins are widely conserved across eukaryotes, from yeasts to plants and mammals. In the genome of *S*. *cerevisiae*, six Sec14-family protein genes, *SEC14* and *SFH1*–*SFH5*, are present. While *SEC14* is an essential gene, *SFH1*–*SFH5* are dispensable for growth. Sfh2–Sfh5 have been reported to possess PITP activities, but not PCTP activities, *in vitro* [32, 33]. The PITP or PCTP activity of Sfh1 was not detected or much lower than that of Sec14 *in vitro* [32–34]. Sfh1 was found to bind to PE when heterologously produced in *Escherichia coli*, and PE in its binding pocket could be replaced with PI or PC *in vitro* [35]. Despite the abilities of the Sec14 family of proteins to transport PI and PC between membranes, their involvement in interorganellar phospholipid transport *in vivo* is still under debate. Rather, due to the abilities of Sec14 to stimulate PI-4-kinase *in vitro* [35], it was proposed that the physiological role of the Sec14 family proteins is to facilitate the production of phosphatidylinositol 4-phosphate (PI4P) by presenting PI to PI-4-kinases [36, 37].

In this study, we isolated and characterized *SFH1* as a multi-copy suppressor of the growth defect of *psd1*Δ on lactate. Our results suggest that overproduction of Sfh1 enhances PE synthesis by Psd2 by means of activating the lipid flux between the ER and endosome.

## Materials and methods

### Media, growth conditions, yeast strains and plasmids

Strains used in this study are listed in Table 1. They were derived from the W303-1A or the BY4741 backgrounds. Gene disruption was performed using auxotrophic marker or drug resistant marker selection, and confirmed by genomic PCR and Southern blotting. Primers and plasmids used in this study are listed in S1 Table and Table 2, respectively. The *psd1*Δ strain was constructed as follows. The DNA fragments of 5’- and 3’-flanking regions of *PSD1* were amplified by PCR using primers PSD1-K1 and delta-PSD1-A-r, and delta-PSD1-B-f, and PSD1-K2, respectively, with the total DNA of W303-1A as a template. These fragments were ligated with a DNA fragment of *ADE2*, which was amplified by PCR using primers ADE2-u and ADE2-l-2 with total DNA of BY4741 as a template, by fusion PCR using primers delta-PSD1-nest-f and delta-PSD1-nest-r to obtain the *PSD1* deletion cassette. The *PSD1* deletion cassette was introduced into W303-1A to obtain *psd1*Δ. *ECT1* deletion cassette was constructed by fusion PCR as follows. The DNA fragments of 5’- and 3’-flanking regions of *ECT1* were amplified by PCR using primers ECT1-delta-f and ECT1-A-r, and ECT1-B-f and ECT1-delta-r, respectively, with the total DNA of W303-1A. DNA fragment of the marker gene, *TRP1*, was amplified by PCR using primers TRP1-u and TRP1-l with pT-TRP1 as a template. These fragments were fused to obtain the *ECT1* deletion cassette. The *ECT1* deletion cassette was introduced into *psd1*Δ to obtain *psd1*Δ*ect1*Δ. *PSD2* deletion cassette was constructed as follows. The DNA fragments of 5’- and 3’-flanking regions of *PSD2* were amplified by PCR using primers PSD2-delta-f and PSD2-A-r, and PSD2-B-f and PSD2-delta-r, respectively, with total DNA of W303-1A as a template. The DNA fragment of the marker gene, *hph*, was amplified by PCR using primers hph-fw and hph-rv with pAG32 plasmid as a template. These fragments were fused to obtain the *PSD2* deletion cassette. The *PSD2* deletion cassette was introduced into *psd1*Δ to obtain *psd1*Δ*psd2*Δ. To delete *SFH1*, a DNA fragment was amplified by PCR using primers SFH1-del-f and SFH1-del-r with total DNA of BY*sfh1*Δ as a template. This fragment was introduced into *psd1*Δ to obtain *psd1*Δ*sfh1*Δ. *PSS1* deletion cassette was constructed as follows. The DNA fragments of 5’- and 3’-franking regions of *PSS1* were amplified by PCR using primers PSS1-del-f and PSS1-LEU2-A-r, and PSS1-LEU2-B-f, and PSS1-del-r, respectively, with the total DNA of W303-1A as a template. These fragments were ligated with a DNA fragment of *LEU2*, which was amplified by PCR using primers LEU2-f and LEU2-r with plasmid YCplac111 as a template, by fusion PCR using primers PSS1-del-f and PSS1-del-r to obtain the *PSS1* deletion cassette. The *PSS1* deletion cassette was introduced into W303-1A to obtain *pss1*Δ. For construction of BY*psd1*Δ, *PSD1* deletion cassette was constructed by fusion PCR using primers PSD1-K1, PSD1-A-r(drug), PSD1-B-f(drug), and PSD1-K2 with total DNA of W303-1A, primers nat1-fw and nat1-rv with pAG25, and primers delta-PSD1-nest-f and delta-PSD1-nest-r for fusion PCR. To delete *MDM34*, a DNA fragment was amplified by PCR using PCR using primers MDM34-del-f and MDM34-del-r with total genome of BY*mdm34*Δ as a template. This fragment was introduced into BY*psd1*Δ or BY*psd1*Δ harboring YCp33-MDM34 to obtain BY*psd1*Δ*mdm34*Δ. BY*psd1*Δ5x-*emc*Δ was constructed as follows. Deletion cassettes of *EMC5* and *EMC6* were amplified by PCR using primers EMC5-del-f, EMC5-del-r, EMC6-del-f, and EMC6-del-r with total DNA of BY*emc5*Δ and BY*emc6*Δ, respectively, and introduced into BY*psd1*Δ and BY4742 to obtain BY*psd1*Δ*emc5*Δ and BY4742*emc6*Δ, respectively. Deletion cassettes of *EMC1* and *EMC3* were constructed by fusion PCR using primers EMC1-del-f, EMC1-del-A-r, EMC1-del-B-f, EMC1-del-r, EMC3-del-f, EMC3-del-A-r, EMC3-del-B-f, and EMC-del-r with total DNA of BY4741, and primers HIS3-f and HIS3-r with pT-HIS3, and introduced into BY*psd1*Δ*emc5*Δ and BY4742*emc6*Δ to obtain BY*psd1*Δ*emc1*Δ*emc5*Δ and BY4742*emc3*Δ*emc6*Δ, respectively. Deletion cassette of *EMC2* was constructed by fusion PCR using primers EMC2-del-f, EMC2-del-A-r, EMC2-del-B-f, and EMC2-del-r with total DNA of BY4741 and primers nat1-fw and nat1-rv with pAG25, and was introduced into BY4742*emc3*Δ*emc6*Δ to obtain BY4742*emc2*Δ*emc3*Δ*emc6*Δ. BY*psd1*Δ5x-*emc*Δ was constructed by mating BY4741*psd1*Δ*emc1*Δ*emc5*Δ and BY4742*emc2*Δ*emc3*Δ*emc6*Δ. BY*psd1*Δ*vps39*Δ was constructed by introducing a DNA fragment amplified by primers VPS39-del-f and VPS39-del-r with total DNA of BY*vps39*Δ into BY*psd1*Δ. To delete *YPT7*, a DNA fragment was amplified by PCR using primers YPT7-del-f and YPT7-del-r with total DNA of BY*ypt7*Δ, and introduced into BY*psd1*Δ to obtain BY*psd1*Δ*ypt7*Δ. For *VPS13* deletion, a DNA fragment was amplified by PCR using primers VPS13-del-f and VPS13-del-r with total DNA of BY*vps13*Δ (EUROSCARF), and introduced into BY*psd1*Δ to obtain BY*psd1*Δ*vps13*Δ. To delete *SFH4*, a DNA fragment was amplified by PCR using primers SFH4-del-f and SFH4-del-r with total DNA of BY*sfh4*Δ, and introduced into *psd1*Δ to obtain *psd1*Δ*sfh4*Δ. For *MMM1*, *MDM10*, and *MDM12* deletion, DNA fragments were amplified by PCR using primers MMM1-del-f, MMM1-del-r, MDM10-del-f, MDM10-del-r, MDM12-del-f and MDM12-del-r with total DNA of BY*mmm1*Δ, BY*mdm10*Δ, and BY*mdm12*Δ, and introduced into BY*psd1*Δ to obtain BY*psd1*Δ*mmm1*Δ, BY*psd1*Δ*mdm10*Δ, and BY*psd1*Δ*mdm12*Δ. To delete *VPS41*, a DNA fragment was amplified by PCR using VPS41-del-f, and VPS41-del-r with total DNA of BY*vps41*Δ, and introduced into BY*psd1*Δ to obtain BY*psd1*Δ*vps41*Δ.

**Table 1:** Strains used in this study.

Name	Genotype	Source
W303-1A	*MATa ade2-1 ura3-1 his3-11*,*15 trp1-1 leu2-3*,*112 can1-100*	ATCC
*psd1*Δ	W303-1A *psd1*Δ::*ADE2*	This study
*psd1*Δ*ect1*Δ	W303-1A *psd1*Δ::*ADE2 ect1*Δ::*TRP1*	This study
*psd1*Δ*psd2*Δ	W303-1A *psd1*Δ::*ADE2 psd2*Δ::*hph*	This study
*psd1*Δ*sfh1*Δ	W303-1A *psd1*Δ::*ADE2 sfh1*Δ::*kanMX4*	This study
*psd1*Δ*sfh4*Δ	W303-1A *psd1*Δ::*nat1 sfh4*Δ::*kanMX4*	This study
*pss1*Δ	W303-1A *pss1*Δ::*LEU2*	This study
BY4741	*MAT*a *his3*Δ*1 leu2*Δ*0 met15*Δ*0 ura3*Δ*0*	EUROSCARF
BY4742	*MAT*α *his3*Δ*1 leu2*Δ*0 lys2*Δ*0 ura3*Δ*0*	EUROSCARF
BY*psd1*Δ	BY4741 *psd1*Δ::*nat1*	This study
*psd1*Δ*mdm34*Δ	BY4741 *psd1*Δ::*nat1 mdm34*Δ::*kanMX4*	This study
*psd1*Δ5x-*emc*Δ	BY4741 *psd1*Δ::*nat1 emc1*Δ::*HIS3 emc2*Δ::*nat1 emc3*Δ::*HIS3 emc5*Δ::*kanMX4 emc6*Δ::*kanMX4*	This study
*psd1*Δ*vps39*Δ	BY4741 *psd1*Δ::*nat1 vps39*Δ::*kanMX4*	This study
*psd1*Δ*ypt7*Δ	BY4741 *psd1*Δ::*nat1 ypt7*Δ::*kanMX4*	This study
*psd1*Δ*vps13*Δ	BY4741 *psd1*Δ::*nat1 vps13*Δ::*kanMX4*	This study
*mmm1*Δ	BY4741 *mmm1*Δ::*kanMX4*	EUROSCARF
*mdm10*Δ	BY4741 *mdm10*Δ::*kanMX4*	EUROSCARF
*mdm12*Δ	BY4741 *mdm12*Δ::*kanMX4*	EUROSCARF
*mdm34*Δ	BY4741 *mdm34*Δ::*kanMX4*	EUROSCARF
*psd1*Δ*mmm1*Δ	BY4741 *psd1*Δ::*nat1 mmm1*Δ::*kanMX4*	This study
*psd1*Δ*mdm10*Δ	BY4741 *psd1*Δ::*nat1 mdm10*Δ::*kanMX4*	This study
*psd1*Δ*mdm12*Δ	BY4741 *psd1*Δ::*nat1 mdm12*Δ::*kanMX4*	This study
*5x-emc*Δ	BY4741 *emc1*Δ::*HIS3 emc2*Δ::*nat1 emc3*Δ::*HIS3 emc5*Δ::*kanMX4 emc6*Δ::*kanMX4*	This study
*vps39*Δ	BY4741 *vps39*Δ::*kanMX4*	EUROSCARF
*ypt7*Δ	BY4741 *ypt7*Δ::*kanMX4*	EUROSCARF
*vps41*Δ	BY4741 *vps41*Δ::*kanMX4*	EUROSCARF
*psd1*Δ*vps41*Δ	BY4741 *psd1*Δ::*nat1 vps41*Δ::*kanMX4*	This study
*vps13*Δ	BY4741 *vps13*Δ::*kanMX4*	EUROSCARF

**Table 2:** Plasmids used in this study.

Plasmid	Descriptions	Reference or source
YEplac181	Multi-copy vector carrying *LEU2*	[38]
YEplac195	Multi-copy vector carrying *URA3*	[38]
YCplac22	Low copy vector carrying *TRP1*	[38]
YCplac33	Low copy vector carrying *URA3*	[38]
YCplac111	Low copy vector carrying *LEU2*	[38]
YEp181-SFH1	YEplac181 carrying *SFH1*	This study
YEp181-SFH2	YEplac181 carrying *SFH2*	This study
YEp181-SFH3	YEplac181 carrying *SFH3*	This study
YEp181-SFH4	YEplac181 carrying *SFH4*	This study
YEp181-SFH5	YEplac181 carrying *SFH5*	This study
YEp181-SEC14	YEplac181 carrying *SEC14*	This study
YEp195-SFH1	YEplac195 carrying *SFH1*	This study
YEp181-SFH1^S175I,T177I^	YEplac181 carrying *sfh1*^*S175I*,*T177I*^	This study
YEp181-SFH1^R61A,T238D^	YEplac181 carrying *sfh1*^*R61A*,*T238D*^	This study
YEp181-SFH1^L179W,I196W^	YEplac181 carrying *sfh1*^*L179W*,*I196W*^	This study
YEp181-SFH1^Y113C^	YEplac181 carrying *SFH1*^Y113C^	This study
YCp111-PSD1	YCplac111 carrying *PSD1*	This study
YEp181-PSD2	YEplac181 carrying *PSD2*	This study
YEp181-DPL1	YEplac181 carrying *DPL1*	This study
YEp181-PSS1	YEplac181 carrying *PSS1*	This study
YCp33-PSS1-EGFP	YCplac33 carrying *PSS1*-*EGFP*	This study
YCp22-PSD2-FLAG	YCplac22 carrying *PSD2*-*FLAG*	This study
pT-TRP1	Plasmid carrying *TRP1*	[39]
pT-HIS3	Plasmid carrying *HIS3*	[39]
pAG32	Plasmid carrying *hph*	[40]
pAG25	Plasmid carrying *nat1*	[40]
YCpTGAP111	Plasmid carrying GAPDH terminator	[41]
pEZZ18	Plasmid carrying *ZZ*	GE healthcare
pEGFP	Plasmid carrying EGFP	Clontech
pFA6a-3HA-kanMX6	Plasmid carrying 3xHA tag	[42]
p3xFLAG-myc-CMVTM-26	Plasmid carrying 3xFLAG tag	Sigma
YEp181-SFH1-ZZ	YEplac181 carrying *SFH1ZZ*	This study
YEp181-SFH1^S175I,T177I^-ZZ	YEplac181 carrying *SFH1*^S175I,T177I^*ZZ*	This study
pET28a	An expression plasmid	Novagen
pETSFH1	A plasmid to express Sfh1 in *E*. *coli*	This study
pETSFH1^S175I,T177I^	A plasmid to express Sfh1 ^S175I,T177I^ in *E*. *coli*	This study
YEp181-SFH1-HA	YEplac181 carrying *SFH1-HA*	This study
YEp181-SFH1-EGFP	YEplac181 carrying *SFH1-EGFP*	This study
YEp181-SFH1^S175I,T177I^-EGFP	YEplac181 carrying *sfh1*^*S175I*,*T177I*^*-EGFP*	This study
YEp181-SFH1^R61A,T238D^-EGFP	YEplac181 carrying *sfh1*^*R61A*,*T238D*^*-EGFP*	This study
YEp181-SFH1^L179W,I196W^-EGFP	YEplac181 carrying *sfh1*^*L179W*,*I196W*^*-EGFP*	This study
YCp33-MDM34	YCplac33 carrying *MDM34*	This study
YCp111-MDM34	YCplac111 carrying *MDM34*	This study

Primers used for the plasmid construction are listed in S1 Table. Plasmids YEp181-SFH1 and YEp195-SFH1 was constructed as follows: A DNA fragment containing ORF and 5’- and 3’-flanking regions of *SFH1* was amplified by PCR using primers SFH1-EcoRI-f and SFH1-SalI-r with total DNA of W303-1A as a template. This fragment was digested with EcoRI and SalI, and cloned into the EcoRI-SalI site of YEplac181 or YEplac195. YEp181-SFH2 was constructed similarly to YEp181-SFH1, using primers SFH2-EcoRI-f and SFH2-SalI-r. YEp181-SFH3, YEp181-SFH4, and YEp181-SFH5 were constructed similarly YEp181-SFH1 using primers SFH3-BamHI-f, SFH3-SalI-r, SFH4-BamHI-f, SFH4-SalI-r, SFH5-BamHI-f, and SFH5-SalI-r with restriction enzymes BamHI and SalI. YEp181-SEC14 was constructed similarly to YEp181-SFH1 using primers SEC14-EcoRI-f and SEC14-PstI-r with restriction enzymes EcoRI and PstI. Plasmid YEp181-SFH1^S175I,T177I^ to overexpress *Sfh1*^*S175I*,*T177I*^ was constructed as follows: DNA fragments were amplified using primers SFH1-EcoRI-f, SFH1-ST-A-r, SFH1-ST-B-f, and SFH1-SalI-r with plasmid YEp181-SFH1 as a template. These fragments were ligated by PCR using SFH1-EcoRI-f and SFH1-SalI-r. Obtained fragment was digested with EcoRI and SalI and cloned into the EcoRI-SalI site of YEplac181. YEp181-SFH1^R61A,T238D^ was constructed similarly to YEp181-SFH1^S175I,T177I^. DNA fragments were amplified by PCR using SFH1-EcoRI-f, SFH1-R61A-r, SFH1-R61A-f, and SFH1-SalI-r with plasmid YEp181-SFH1 as a template. These fragments were ligated by PCR using SFH1-EcoRI-f and SFH1-SalI-r. This fragment was used as a template for PCR using primers SFH1-EcoRI-f, SFH1-T238D-r, SFH1-R61A-f, and SFH1-SalI-r to obtain two DNA fragments. These fragments were ligated by PCR using SFH1-EcoRI-f and SFH1-SalI-r. Obtained fragment was digested with EcoRI and SalI, and cloned into the EcoRI-SalI site of YEplac181. YEp181-SFH1^L179W,I196W^ was constructed similarly to YEp181-SFH1^R61A,T238D^, using primers SFH1-EcoRI-f, SFH1-L179W-r, SFH1-L179W-f, SFH1-I196W-r, SFH1-I196W-f, and SFH1-SalI-r. YEp181-SFH1^Y113C^ was constructed similarly to YEp181-SFH1^S175I,T177I^ using primers SFH1-EcoRI-f, SFH1-Y113C-r, SFH1-Y113C-f, and SFH1-SalI-r.

Plasmids YEp181-SFH1-ZZ and YEp181-SFH1^S175I,T177I^-ZZ to express Sfh1-ZZ or Sfh1^S175I,T177I^-ZZ were constructed as follows: DNA fragments were amplified by PCR using primers SFH1-EcoRI-f, SFH1ctag-SacI-r, ZZ-SacI-f, and ZZ-SalI-r with plasmid YEp181-SFH1, YEp181-SFH1^S175I,T177I^, or pEZZ18 as templates. These fragments were digested with EcoRI and SacI, or SacI and SalI, respectively. YCpTGAP111 was digested with SalI and HindIII to obtain DNA fragment of *TDH1* terminator. These fragments were cloned into the EcoRI-HindIII site of YEplac181. Plasmids YEp181-SFH1-EGFP and YEp181-SFH1-HA to express C-terminally tagged with EGFP or 3xHA epitope were constructed similarly to YEp181-SFH1-ZZ. DNA fragments of EGFP or 3xHA were amplified by PCR using primers EGFP-SacI-f and EGFP-SalI-r, or 3xHA-SacI-f and 3xHA-SalI-r with pEGFP or pFA6a-3HA-kanMX6 as templates. Plasmids pETSFH1 and pETSFH1^S175I,T177I^ to express N-terminally His_8_-tagged Sfh1 or Sfh1^S175I,T177I^ in *E*. *coli* were constructed as follows: DNA fragments were amplified by PCR using primers His8-SFH1-fw and His8-SFH1-rv with plasmids YEp181-SFH1 or YEp181-SFH1^S175I,T177I^ as templates. These fragments were digested with NcoI and SacI, and cloned into the NcoI-SacI site of pET28a.

Plasmid YCp111-PSD1 to express *PSD1* under the control of its native promoter was constructed as follows: A DNA fragment containing ORF with 5’- and 3’-flanking regions of *PSD1* was amplified by PCR using primers PSD1-KpnI-f and SFH1-BamHI-r with total DNA of W303-1A as a template. This fragment was digested with KpnI and BamHI and cloned into the KpnI-BamHI site of YCplac111. Plasmids YEp181-PSD2, YEp181-DPL1, and YEp181-PSS1 to overexpress *PSD2*, *DPL1*, or *PSS1* were constructed as follows: DNA fragment containing ORF with 5’- and 3’-flanking regions of *PSD2*, *DPL1*, or *PSS1* was amplified by PCR using primers PSD2-HindIII-f and PSD2-SalI-r, PstI-DPL1-f and DPL1-SacI-r, or PstI-PSS1-f, and PSS1-SacI-r, respectively. These fragments were digested by HindIII and SalI, or PstI and SacI and cloned into the HindIII-SalI site or PstI-SacI site of YEplac181. Plasmid YCp33-PSS1-EGFP to express Pss1-EGFP fusion protein under the control of its native promoter was constructed as follows: DNA fragments were amplified by PCR using primers BamHI-PSS1-f and PSS1ctag-KpnI-r, or KpnI-EGFP-f and EGFP-SalI-r with total DNA of W303-1A or plasmid pEGFP as a template, respectively. These fragments were digested by BamHI and KpnI or KpnI and SalI. YCpTGAP111 was digested with SalI and HindIII to obtain DNA fragment of *TDH1* terminator. These three digested fragments were cloned into the BamHI-HindIII site of YCplac33. YCp22-PSD2-FLAG to express Psd2-FLAG fusion protein was constructed as follows: DNA fragment was amplified by PCR using primers PSD2-HindIII-f and PSD2ctag-SalI-r, SalI-FLAG-f and FLAG-GAPDHt-r, or FLAG-GAPDHt-f and GAPDHt-SpeI-r with total DNA of W303-1A, p3xFLAG-myc-CMVTM-26, or YCpTGAP111 as a template, respectively. The DNA fragments containing FLAG and *TDH1* terminator were ligated by PCR using primers SalI-FLAG-f and GAPDHt-SpeI-r. This fragment and *PSD2* fragment were digested by SalI and SpeI or HindIII and SalI, and cloned into the HindIII-SpeI site of YCplac22. Plasmids YEp195-MDM34 and YCp111-MDM34 to express MDM34 under the control of its native promoter were constructed as follows: A DNA fragment was amplified by PCR using primers SalI-MDM34-f and HindIII-MDM34-r with total DNA of BY4741 as template. This fragment was digested with SalI and HindIII, and cloned into the SalI-HindIII site of YEplac195 or YCplac111. Yeast cells were grown on minimal medium (0.17% yeast nitrogen base without amino acid and ammonium sulfate, 0.5% ammonium sulfate) containing 2% glucose (SD medium) or 4.4% lactate (pH 5.5) (SLac medium), or semi-synthetic lactate medium (4.4% lactate, 1.6% NaOH, 0.17% yeast nitrogen base without amino acid and ammonium sulfate, 0.5% ammonium sulfate, 0.3% yeast extract, 0.05% glucose, 0.05% CaCl_2_·2H_2_O, 0.05% NaCl, 0.06% MgCl_2_·6H_2_O, 0.1% NH_4_Cl, 0.1% KH_2_PO_4_, pH 5.5), with required nutrients at 30°C. For growth curve analyses, cells precultured in SD medium were seeded at a starting OD_600_ = 0.005 to the medium of interest. Cells were cultured at 30°C and the growth curve was obtained with an automatically recording incubator TN1506 (Advantec).

### Lipid analysis

For analyzing phospholipid composition, cells were grown in SD medium or semi-synthetic lactate medium to a final OD_600_ between 1.0 and 2.0, harvested by centrifugation, and washed with ice-cold 0.15 M KCl. Cells were broken with glass beads in chloroform/methanol/water (2:4:1). For analysis of mitochondrial phospholipids, yeast spheroplasts were lysed gently by French pressure cell press (SLM instruments), and mitochondria were purified by sucrose density gradient centrifugation by the method of Zinser and Daum [1]. Total lipids were extracted from organic layer by the method of Bligh and Dyer [43]. The extracted lipids were separated by two-dimensional thin layer chromatography (TLC) as previously described [44]. The spots corresponding to phospholipids were scraped from TLC plates. Phosphorus assay was done for each of phospholipid species according to the method of Bartlett [45].

For the analysis of Sfh1-binding lipids, W303-1A expressing Sfh1-ZZ, Sfh1^S175I,T177I^-ZZ, or non-tagged Sfh1 were grown in SD medium to a final OD_600_ between 1.5 and 2.0, and harvested by centrifugation. Cells were suspended in Lysis buffer (25 mM HEPES (pH 7.5), 100 mM KCl, 10%(w/v) Glycerol, 1 mM DTT, Protease Inhibitor Cocktail for Fungal and Yeast cells (Sigma)), and disrupted with glass beads using Multi-Beads Shocker (Yasui Kikai). Cytosolic fractions were obtained by stepwise centrifugation at 13,000 g for 10 min at 4°C, and at 100,000 g for 60 min at 4°C twice (S100 fraction). The S100 fractions (4 mg protein) were incubated with IgG-Sepharose^TM^6 Fast Flow (GE Healthcare) beads overnight at 4°C. Beads were washed with ice-cold lysis buffer and lipids were extracted from the beads by the method of Bligh and Dyer with short-chain phospholipids, diC10 PC, diC8 PE, diC8 PI, or diC12 PS (Avanti Polar Lipids) (0.35 nmol each) for internal standard. Phospholipid species were analyzed by ESI-MS/MS according to the method previously described [44, 46, 47]. For ionization efficiency correction, mixture of equal molar of short-chain phospholipids used for internal standards with DOPC, DOPE, SoyPI, or DOPS was subjected to ESI-MS/MS analysis. Ionization rates of DOPC, DOPE, SoyPI, and DOPS against short-chain phospholipids were 92 ± 5.5%, 22 ± 1.0%, 23 ± 0.7%, and 26 ± 5.6%, respectively, and employed as correction factors.

### Protein expression and purification

Recombinant His_8_-Sfh1 and His_8_-Sfh1^S175I,T177I^ mutant were expressed in *Escherichia coli* BL21 (DE3) strain and purified as described previously [48] with several modifications. *E*. *coli* cells were lysed by French pressure cell press in lysis buffer containing 25 mM HEPES (pH7.5), 300 mM KCl, 10% (w/v) Glycerol, Protease Inhibitor Cocktail for use in Histidine-tagged proteins purification (Sigma), and 2 mM β-mercaptoethanol. Cell extracts were incubated with Talon Metal Affinity Resin (clontech) beads overnight at 4°C. Beads were washed with lysis buffer and lysis buffer containing 5 mM imidazole. Proteins were eluted by lysis buffer containing 200 mM imidazole. Buffer exchange against lysis buffer was performed using PD-10 desalting column (Amersham Biosciences).

### Determination of Pss1 activity

Pss1 activity was analyzed by the method of Bae-Lee and Carman [5] and Kiyono *et al*. [49] with some modifications. ER fraction of *psd1*Δ*psd2*Δ was purified by the method previously described [50]. ER fraction and recombinant His_8_-Sfh1 were diluted to 2 mg/mL by dilution buffer containing 25 mM HEPES-KOH (pH 7.5), 100 mM KCl, and 10% (w/v) glycerol. Sixty-three μL of premix containing 25 mM HEPES-KOH (pH7.5), 100 mM KCl, 1 mM MgCl_2_, 0.6 mM MnCl_2_, 0.4 mM CTP, 8.5 mM β-chloroalanine, 10% (w/v) glycerol, and 2.5 mM L-serine was mixed with 2 μL (1 μCi/μL) [^3^H] serine. Mixture of 10 μL dilution buffer, 5 μL His_8_-Sfh1, and 20 μL ER fraction were added and incubated at 30°C for 15, 30, 45, and 60 min. The reaction was terminated by the addition of 0.5 mL methanol acidified with 0.1 N HCl and 1.5 mL chloroform. Water-soluble materials were removed by repeated washing with 3 mL 1 M MgCl_2_. The radioactivity in the chloroform layer was analyzed by liquid scintillation counter.

### Determination of Psd2 activity

Psd2 activity was analyzed by the method of Trotter and Voelker [9] with some modifications. Cells were grown in SD medium to final OD_600_ between 1.0 and 1.5 and harvested by centrifugation. Cells were suspended in Lysis buffer (10 mM Potassium phosphate (pH 7.4), 0.25 M Sucrose, 3 mM EDTA, 1 mM DTT, Protease Inhibitor Cocktail for use in Histidine-tagged protein purification), and disrupted with glass beads using Multi-bead shocker. Postnuclear supernatants (PNSs) were prepared by centrifugation of the cell lysates at 1,000 × g for 10 min at 4°C twice. Lipid film of 16:0–6:0 NBD-PS (1-palmitoyl-2-(6-((7-nitro-2-1,3-benzoxadiazol-4-yl)amino)hexanoyl)-*sn*-glycero-3-phosphoserine) (Avanti Polar Lipids, Inc.) was dissolved in reaction buffer (25 mM KH_2_PO_4_ (pH 7.4), 0.125 M Sucrose, 1 mM EDTA) to final concentration of 10 μM. PNSs (10 μg protein) were added to the reaction buffer containing NBD-PS to bed volume of 400 μL and incubated for 0 or 15 min at 36°C. Reaction was stopped by addition of 2.4 mL chloroform/methanol (1:2, v/v). Total lipids were extracted by the method of Bligh and Dyer and separated by TLC using chloroform/methanol/acetone/acetate/H_2_O (100:20:40: 30: 10, v/v/v/v/v) as a developing solvent. The spots corresponding to NBD-phospholipids were scraped from TLC plates, and lipids were extracted with methanol. NBD fluorescence of the extracted lipids was measured by SH-8000 (CORONA ELECTRIC Co., Ltd.). Values of NBD fluorescence of time point = 0 samples were subtracted for background correction.

### Fluorescence dequenching assay

Fluorescent based phospholipid transfer assay was carried out as described previously [51], with some modifications. The phospholipids DOPC (1,2-dioleoyl-*sn*-glycero-3-phosphocholine), DOPE (1,2-dioleoyl-*sn*-glycero-3-phosphoethanolamine), DOPS (1,2-dioleoyl-*sn*-glycero-3-phosphoserine), soyPI (L-α-phosphatidylinositol (Soy)), 16:0–6:0 NBD-PS (1-palmitoyl-2-{6-[(7-nitro-2-1,3-benzoxadiazol-4-yl)amino]hexanoyl}-*sn*-glycero-3-phosphoserine), 16:0–6:0 NBD-PC (1-palmytoyl-2-{6-[(7-nitro-2-1,3-benzoxadiazol-4-yl)hexanoyl]-sn-glycero-3-phosphocholine}, 14:0–6:0 NBD-PE (1-myristoyl-2-{6-[(7-nitro-2-1,3-benzoxadiazol-4-yl)amino]hexanoyl}-*sn*-glycero-3-phosphoethanolamine), and 18:1 Liss-Rhod-PE (1,2-dioleoyl-*sn*-glycero-3-phosphoethanolamine-N-(lissamine rhodamine B sulfonyl) (ammonium salt)) were purchased from Avanti Polar Lipids, Inc.. Donor liposomes (DOPC:DOPE:NBD-phospholipids:Rhod-PE in a 68:22:8:2 mol ratio) and Acceptor liposome (DOPC:DOPE:soyPI:DOPS in a 50:10:25:15 mol ratio) were prepared by hydration of lipid films with assay buffer (20mM Tris-HCl pH 7.5, 150mM NaCl and 2mM EDTA), freeze-thawing for 5 times, and extrusion for 21 strokes through polycarbonate 0.1-mm filter (AVESTIN). Donor and acceptor liposomes were mixed to final concentration of 25 μM and 250 μM on ice, and His_8_-Sfh1, His_8_-Sfh1^S175I,T177I^, recombinant lactate dehydrogenase from rabbit muscle (rLDH) (Oriental Yeast Co., LTD) or buffer was added to bed volume of 200 μL. The NBD fluorescence (Ex; 460 nm, Em; 534 nm) was monitored at room temperature by SH-9000 (CORONA ELECTRIC Co., Ltd.).

### Fluorescence microscopic observation

Cells were cultured in the SD medium to logarithmic phase and fluorescence was detected by Olympus BX-52 microscope equipped with a digital CCD camera ORCA-ER 95-4742-ER (Hamamatsu Photonics) and an imaging system AQUACOSMOS (Hamamatsu photonics).

### Fractionation of cell extract

Fractionation was performed as described previously [41]. Anti-Pgk1 (Molecular probes), anti-Pep12 (Molecular probes), anti-Sed5 (provided by Koji Yoda), anti-CPY (provided by Akihiko Nakano), anti-Dpm1 (Molecular probes), and anti-Cox2 (Invitrogen) antibodies were used for organelle markers for the cytosol, endosome, Golgi apparatus, vacuole, endoplasmic reticulum, and mitochondria, respectively.

## Results

### Suppression of Etn auxotrophy of *psd1*Δ on non-fermentable carbon sources by *SFH1* overexpression

**Fig 2:**
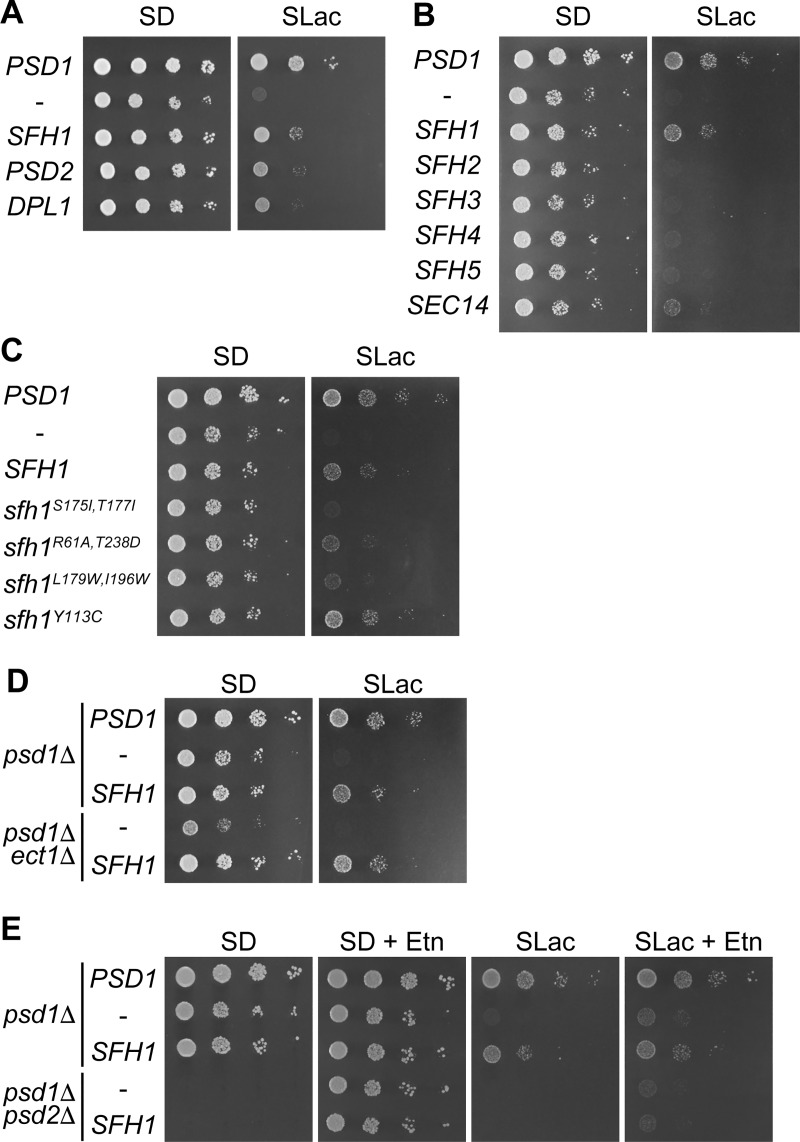
Overexpression of *SFH1* restores the growth of the *psd1*Δ strain on lactate. Cells were cultured in SD medium to logarithmic phase and were spotted on SD or SLac media in ten-fold serial dilutions and were incubated on SD medium for 2 days or on SLac medium for 7 days. (A) Growth of the *psd1*Δ strains overexpressing genes obtained from the screening. (B) Growth of *psd1*Δ strains overexpressing genes encoding Sec14 family proteins, Sec14 and Sfh1–Sfh5. (C) Growth of the *psd1*Δ strains overexpressing *SFH1* mutants, *sfh1*^*S175I*,*T177I*^, *sfh1*^*R61A*,*T238D*^, *sfh1*^*L179W*,*I196W*^, and *sfh1*^*L179W*,*I196W*^. (D) Growth of *psd1*Δ*ect1*Δ overexpressing *SFH1*. (E) Growth of *psd1*Δ*psd2*Δ overexpressing *SFH1*. Etn was added to final concentration of 1 mM.

The *PSD1* deletion mutant showed a significant defect in growth on non-fermentable carbon sources, but supplementation of Etn suppressed this defect [52], suggesting that PE synthesized in other organelle(s) is transported to the mitochondria. To identify the genes involved in PE supply to the mitochondria in *S*. *cerevisiae*, we carried out genetic screening for multi-copy suppressors of Etn auxotrophy of *psd1*Δ on lactate. A multi-copy genomic library was introduced into *psd1*Δ and clones that restored the growth on synthetic lactate (SLac) medium in the absence of Etn supplementation were isolated. These clones are expected to increase the amount of mitochondrial PE by enhancing PE synthesis or PE import to the mitochondria. Indeed, genes involved in PE synthesis were obtained from this screening; overexpression of *PSD2* and *DPL1*, which encodes a dihydrosphingosine phosphate lyase involved in the synthesis of PE via the Kennedy pathway, partially recovered the growth of *psd1*Δ on SLac (Fig 2A). After screening approximately 12,500 transformants, we isolated a clone containing *SFH1/YKL091c* encoding a member of the Sec14 family proteins. Overexpression of *SFH1* by a multi-copy vector suppressed the growth defect of *psd1*Δ on lactate more efficiently than *PSD2* or *DPL1* (Fig 2A). *SFH1* overexpression also restored the growth of *psd1*Δ on the medium containing other non-fermentable carbon sources, including glycerol and ethanol (S1A Fig).

Overexpression of *SEC14* weakly suppressed the growth defect of *psd1*Δ on lactate, but overexpression of other Sec14-family protein genes, including *SFH4*, which is critical for Psd2 activity *in vivo* [10], did not (Fig 2B). Previous studies have indicated that the endogenous Sec14 level was more than 20-fold higher than that of Sfh1 in wild-type *S*. *cerevisiae* cells cultured in the medium containing glucose [53, 54]. Therefore, it is conceivable that the suppressor function of Sec14 is much weaker than that of Sfh1 and that the suppression of the growth defect of *psd1*Δ on non-fermentable carbon sources is a unique function of Sfh1 amongst the yeast Sec14-family proteins. The Y113C substitution confers Sec14-like functions in vesicle transport to Sfh1 [34], but did not abolish the activity of Sfh1 in suppressing the growth defect of *psd1*Δ on lactate (Fig 2C).

Crystal structure analysis revealed that recombinant Sfh1 binds PI, PC, and PE in its hydrophobic pocket [35]. Sec14 is reported to possess PITP and PCTP activities *in vitro* [31], but both the PITP and PCTP activities of Sfh1 are approximately fivefold lower than those of Sec14 [34]. Sfh1^S175I,T177I^, Sfh1^R61A,T238D^, and Sfh1^L179W,I196W^ mutants are reported to be defective in binding to PC/PE, PI, and PC/PE/PI, respectively [35]. Overexpression of *Sfh1*^*S175I*,*T177I*^ and *sfh1*^*L179W*,*I196W*^ mutant genes did not suppress the growth defect of *psd1*Δ on lactate while overexpression of *sfh*^*R61AT238D*^ partially restored the growth of *psd1*Δ (Fig 2C). We analyzed the levels of Sfh1 mutant proteins tagged with EGFP, whose overproduction showed similar effects with Sfh1 mutants without EGFP tag on the growth of *psd1*Δ, and expression levels of these Sfh1 mutants tagged with EGFP were found to be not less than that of the wild-type Sfh1 tagged with EGFP (S2 Fig), suggesting that these mutants were overexpressed. These results imply that PC/PE binding (and probably transport) is a prerequisite for Sfh1 function whereas PI binding (and probably transport) is partially dispensable.

In the *psd1*Δ cells, there remain two routes to synthesize PE: the Kennedy pathway in the ER and PS decarboxylation by Psd2 in the endosome, Golgi, and/or vacuole [52]. To clarify the contribution of these routes to the Sfh1-mediated growth recovery on lactate, we validated the growth of the double deletion mutant of *PSD1* and *ECT1*, which encodes ethanolamine-phosphate cytidylyltransferase, a critical enzyme in the PE synthesis through the Kennedy pathway, and that of *PSD1* and *PSD2* or *SFH4* in the presence of *SFH1* overexpression. Overexpression of *SFH1* restored the growth of *psd1*Δ*ect1*Δ on lactate, suggesting that the Kennedy pathway is dispensable for the suppressor ability of *SFH1* (Fig 2D). In contrast, *SFH1* overexpression did not recover the growth defect of *psd1*Δ*psd2*Δ or *psd1*Δ*sfh4*Δ on lactate (Fig 2E and S1B Fig), revealing that PE synthesized by Psd2 is critical for the suppression of the growth defect of *psd1*Δ on lactate by the overexpression of *SFH1*. In accordance with this result, simultaneous overexpression of *SFH1* and *PSD2* improved the growth of *psd1*Δ on lactate compared with overexpression of each gene alone (S1C Fig).

### Restoration of mitochondrial function of *psd1*Δ by *SFH1* overexpression

**Fig 3:**
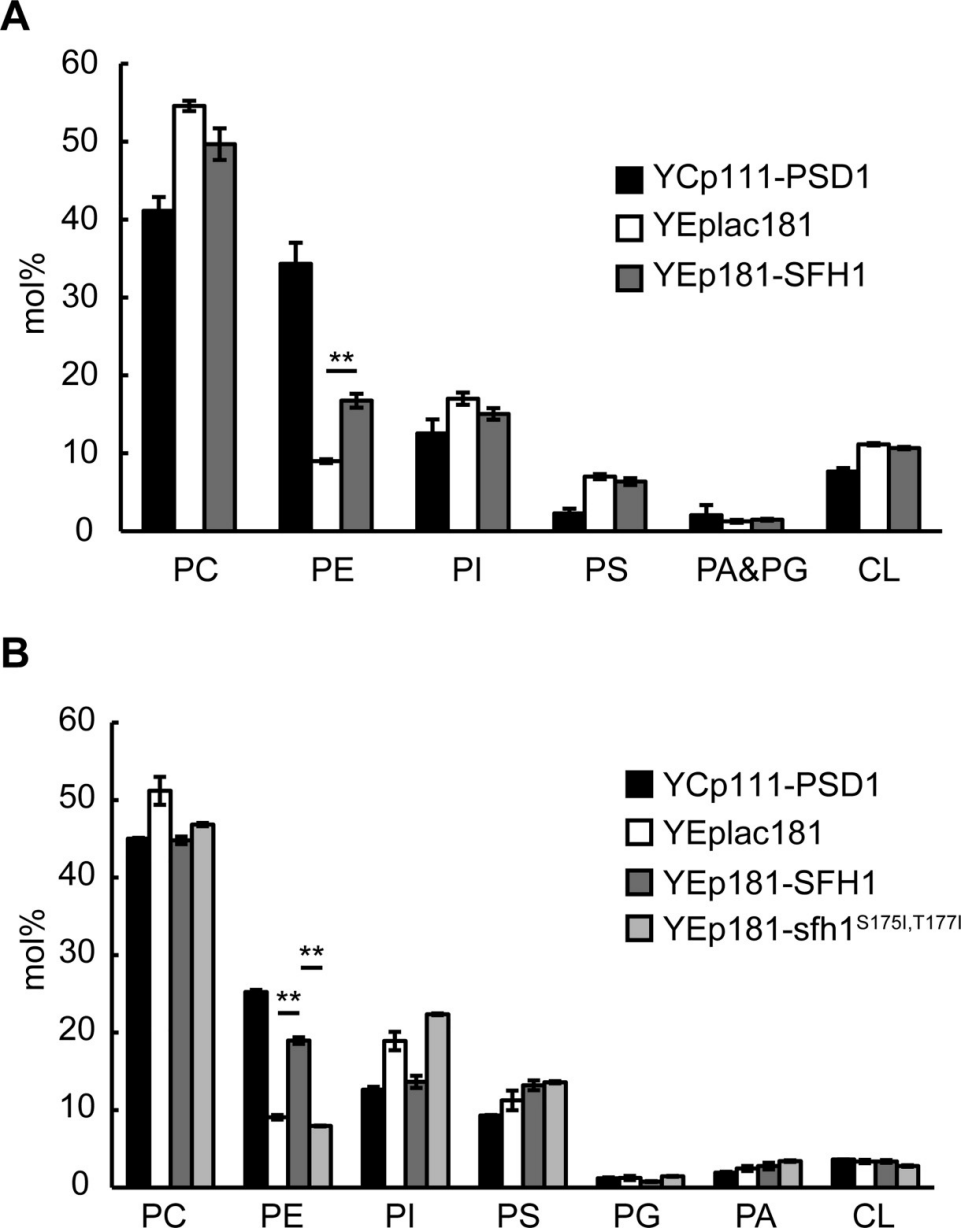
Overexpression of *SFH1* restores the phospholipid compositions and MRC IV protein level of the *psd1*Δ strain. **(**A) Mitochondrial phospholipid composition of *psd1*Δ overexpressing *SFH1*. The *psd1*Δ strains harboring YCp111-PSD1, YEplac181, or YEp181-SFH1 were cultured in semi-synthetic lactate medium to late logarithmic phase, and mitochondria were purified by sucrose density gradient. Lipids were extracted by the Bligh and Dyer’s method, separated by thin-layer chromatography, and analyzed as described in the Material and Methods. Data are the means of three independent assays. Error bars represent S.E. **, p < 0.005 (two-tailed Student’s t-test) (B) Cellular phospholipid composition of *psd1*Δ overexpressing *SFH1*. The *psd1*Δ strains harboring YCp111-PSD1, YEplac181, YEp181-SFH1, or YEp181-SFH1^S175I,T177I^ were cultured in semi-synthetic lactate medium to late logarithmic phase. Lipids were extracted and analyzed as described above. Data are the means of three independent assays. Error bars represent S.E. **, p < 0.005 (two-tailed Student’s t-test).

To determine whether overexpression of *SFH1* increased the mitochondrial PE level of *psd1*Δ, we purified the mitochondrial fraction from *psd1*Δ in the presence or absence of *SFH1* overexpression and analyzed the phospholipid composition. Deletion of *PSD1* caused a three-fold reduction in the mitochondrial PE level, and overexpression of *SFH1* in *psd1*Δ partially restored it (Fig 3A).

We next analyzed the cellular phospholipid composition of *psd1*Δ with and without *SFH1* overexpression. The *psd1*Δ cellular PE level increased following *SFH1* overexpression (Fig 3B), while the cellular PE level of *psd1*Δ*psd2*Δ did not (S3 Fig), suggesting that overexpression of *SFH1* leads to an increase in PE synthesized by Psd2. Moreover, the fact that overexpression of the *Sfh1*^*S175I*,*T177I*^ mutant did not increase the cellular PE level (Fig 3B) agreed with the inability of this mutant to restore the growth of *psd1*Δ on lactate (Fig 2C).

**Fig 4:**
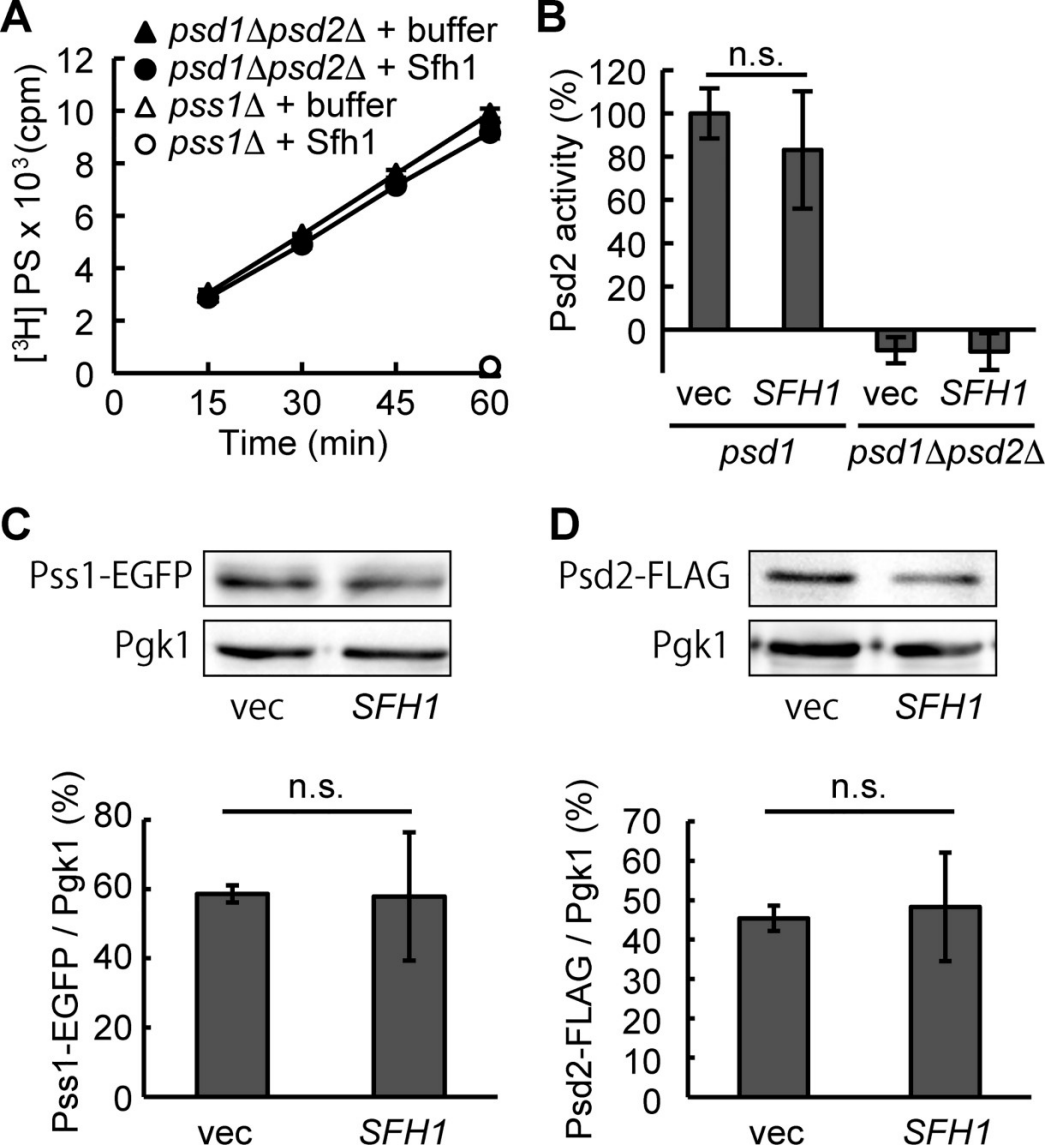
*SFH1* does not activate or increase Pss1 or Psd2 of the *psd1*Δ strain. (A) Addition of recombinant Sfh1 did not potentiate Pss1 activity *in vitro*. Purified ER fraction from *psd1*Δ*psd2*Δ (closed symbols) or *pss1*Δ (open symbols) was mixed with [^3^H]L-serine in the presence (circles) or absence (triangles) of His_8_-Sfh1, and incubated at 30°C for 15, 30, 45, and 60 min. Pss1 activity was measured as described in Materials and methods. Data are the means of three independent assays. Error bars represent S.E. Note that the symbol of the Pss1 activity of the *pss1*Δ in the absence of His_8_-Sfh1 is close to that of the *pss1*Δ in the presence of His_8_-Sfh1. (B) Overexpression of *SFH1* did not potentiate Psd2 activity. Postnuclear supernatant (PNS) (10 μg protein) prepared from *psd1*Δ or *psd1*Δ*psd2*Δ harboring *SFH1* overexpression plasmid or empty vector was mixed with reaction solution containing 16:0–6:0 NBD-PS (10 μM) and incubated at 36°C for 15 min. Lipids were extracted by the Bligh and Dyer’s method and separated by TLC. NBD-PS was quantitated as described in Materials and methods. Data are the means of three independent assays. Error bars represent S.E. n.s., not significant. (C) and (D) Overexpression of *SFH1* did not increase the protein levels of Pss1 and Psd2. Pss1-EGFP (C) and Psd2-FLAG (D) were expressed by low-copy vectors in *psd1*Δ harboring *SFH1* overexpression plasmid or an empty vector. Protein levels were evaluated by immunoblot utilizing anti-GFP, anti-FLAG, and anti-Pgk1 antibodies. Image analysis of immunoblots signal intensities were carried out using Image J. Data are the means of three independent assays. Error bars represent S.E. n.s., not significant.

A possible reason for the elevated cellular PE level by *SFH1* overexpression is that Sfh1 directly activates the enzyme involved in the synthesis of PE or its precursor PS. To test this possibility, we analyzed the activity of Pss1 and Psd2 *in vitro*. However, the addition of the recombinant His_8_-Sfh1 did not increase the activity of Pss1 in the isolated ER fraction (Fig 4A). In addition, overexpression of *SFH1* had no significant effect on Psd2 activity in the *psd1*Δ cell extract (Fig 4B). Furthermore, *SFH1* overexpression did not significantly increase the amount of epitope-tagged Pss1 and Psd2 proteins, which were expressed from the low copy plasmids under their native promoters (Fig 4C and 4D and S4 Fig).

### Binding and transfer of phospholipids by Sfh1

**Fig 5:**
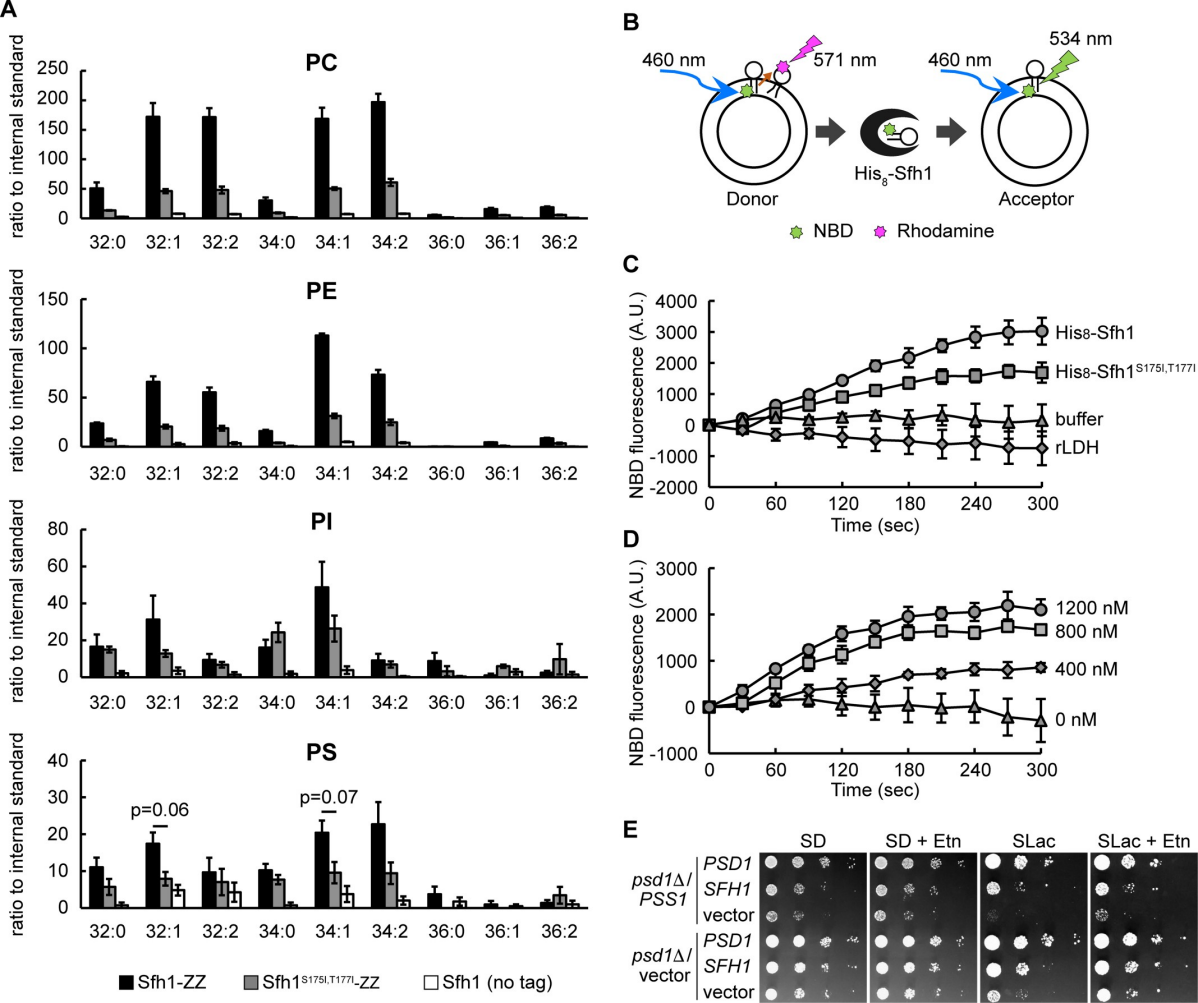
Sfh1 binds phospholipids *in vivo* and transfers PS between liposomes *in vitro*. (A) Phospholipids bound to Sfh1-ZZ and Sfh1^S175I,T177I^-ZZ. The wild-type strains harboring YEp181-SFH1-ZZ, YEp181-SFH1^S175I,T177I^-ZZ or YE181-SFH1 were cultured in SD medium to logarithmic phase. Proteins were purified from S100 fractions (4 mg protein) prepared from these cells. Co-purified lipids were extracted and analyzed by ESI-MS/MS. Data are the means of three independent assays. Error bars represent S.E. p values of two-tailed Student’s t-test are shown. (B) A schematic diagram of fluorescent dequenching assay. See Materials and Methods for details. (C) and (D) NBD-PS dequenching assay by Sfh1 and Sfh1^S175I,T177I^ mutant were measured. Donor liposome (DOPC:DOPE:NBD-PS:Rhod-PE = 68:22:8:2 (mol%)) and acceptor liposome (DOPC:DOPE:soyPI:DOPS = 50:10:25:15 (mol%)) were mixed to final concentration of 25 μM and 250 μM, respectively. Fluorescence of NBD were chased at room temperature immediately after the addition of proteins or buffer. NBD fluorescence intensities were set to 0 at 0 s. Data are the means of three independent assays. Error bars represent S.E. (C) Proteins were added to final concentration of 800 nM. (D) NBD-PS dequenching assay by Sfh1 of various concentration (0, 400, 800, and 1200 nM). (E) Simultaneous *SFH1* overexpression recovers the growth of *psd1*Δ overexpressing *PSS1* on lactate. Cells were cultured in SD medium to logarithmic phase, and were spotted on SD or SLac medium in ten-fold serial dilutions and were incubated on SD medium for 2 days or on SLac medium for 7 days.

Another possibility for the elevated PE level by *SFH1* overexpression is that Sfh1 mediates interorganellar transfer of PS and/or PE and its overproduction enhanced PE synthesis by Psd2 through increased PS supply and/or efficient removal of PE. To determine whether Sfh1 binds to PS and/or PE *in vivo*, Sfh1 was purified from the cytosolic fraction of yeast cell extracts and the lipid species recovered with Sfh1 were analyzed. To accomplish this, Sfh1 was fused with an IgG binding domain (ZZ tag) at its C-terminus (Sfh1-ZZ) and this protein was overproduced in *psd1*Δ. This fusion protein was confirmed to be functional, because its overproduction suppressed the growth defect of *psd1*Δ on lactate (S5A Fig). Sfh1-ZZ was then affinity-purified from the cytosolic fraction (S5B Fig), and the phospholipids bound to Sfh1-ZZ were analyzed by electrospray ionization tandem mass spectrometry (ESI-MS/MS). Significant amounts of PC, PE, PI, and PS were recovered in the extract prepared from Sfh1-ZZ, whereas much smaller amounts of these phospholipids were detected in the fraction similarly prepared from the cells overexpressing the non-tagged Sfh1 (Fig 5A), excluding the possibility of non-specific binding of lipids to the IgG sepharose beads used for protein purification. Compared with phospholipids detected from Sfh1-ZZ, significantly lower amounts of PE and PC were recovered from Sfh1^S175I,T177I^-ZZ, in agreement with the defect in PE/PC binding of recombinant Sfh1^S175I,T177I^. Furthermore, the amount of PS recovered from Sfh1^S175I,T177I^-ZZ appeared to be lower than that from Sfh1-ZZ (C32:1 and C34:1, *p* = 0.06), raising the possibility that Ser 175 and Thr 177 are also important for PS binding. Together, the ability of Sfh1 to bind aminophospholipids was suggested to be critical for its function. Maeda *et al*. have analyzed the lipids bound to Sfh proteins and showed that Sfh1 binds PI and PC, but not PS and PE, *in vivo* [55]. The reason for this discrepancy remains unclear, but it might be due to the difference in the amounts of phospholipids that bind to Sfh1. Alternatively, it may be explained by the difference in the experimental conditions.

We next investigated whether Sfh1 possesses the ability to transfer aminophospholipids between liposomes *in vitro* by a fluorescent dequenching assay. Donor liposome containing 7-nitro-2-1,3-benzoxadiazol-4-yl (NBD)-phospholipid and rhodamine-PE were incubated with recombinant proteins and acceptor liposome mimicking the phospholipid composition of *psd1*Δ cells. While the NBD fluorescence is quenched by rhodamine when NBD-phospholipid and rhodamine-PE are on the same liposome, it is detectable when NBD-phospholipid is transferred to the rhodamine-free liposome (Fig 5B). The addition of recombinant Sfh1 dequenched the fluorescence of NBD-PS in the transfer assay (Fig 5C). No dequenching was observed when the acceptor liposome was absent in the reaction mixture (S5C Fig). Importantly, dequenching of NBD-PS by Sfh1^S175I,T177I^ mutant was less efficient than the wild-type Sfh1. Addition of non-LTP protein (LDH, lactate dehydrogenase) showed no increase in NBD fluorescence, indicating that liposome fusion by non-specific proteins did not occur in this condition [56]. Dequenching of the NBD-PS fluorescence by Sfh1 was observed in a dose-dependent manner (Fig 5D). These results suggest that Sfh1 has the ability to transfer NBD-PS between liposomes *in vitro*. The fluorescence of NBD-PC and NBD-PE declined in a time-dependent manner for an unknown reason, but the decrease in the fluorescence was significantly alleviated by the addition of the wild-type Sfh1 (S5D and S5E Fig). The addition of the Sfh1^S175I,T177I^ mutant showed the same effects. It is also possible that Sfh1 transferred non-labeled phospholipids from the acceptor liposome to the donor liposome, resulting in dilution of the fluorescent phospholipids, which culminates in dequenching of NBD fluorescence. However, because significant increase in the dequenching signal by Sfh1 was detected when NBD-PS, but not NBD-PE or NBD-PC, was used, it is plausible that NBD-PS was transported from the donor liposome to the acceptor liposome.

While overexpression of *PSD2* recovered the growth of *psd1*Δ on lactate (Fig 2A), overexpression of *PSS1* impaired it (Fig 5E), implying that an excess of PS in the *psd1*Δ mitochondria may impair mitochondrial function. Simultaneous overexpression of *SFH1*, however, recovered the growth of *psd1*Δ overexpressing *PSS1* (Fig 5E). Enhanced transport of PS from the ER to endosome, Golgi, and/or vacuole by *SFH1* overexpression and the following conversion of PS to PE by Psd2 may have reduced the mitochondrial PS level. We analyzed the cellular phospholipid levels in *psd1*Δ, *psd1*Δ overexpressing *PSS1*, and *psd1*Δ overexpressing *PSS1* and *SFH1* (S6 Fig). However, *PSS1* overexpression did not significantly increase the cellular PS level, and simultaneous overexpression of *SFH1* did not decrease it. It is possible that *PSS1* overexpression resulted in increased the local PS levels of mitochondria or other organelles and simultaneous overexpression of *SFH1* decreased it. Overexpression of *SFH4* did not recover the growth of *psd1*Δ overexpressing *PSS1* (S5F Fig).

### Localization of Sfh1 to the cytosol and organellar membranes

**Fig 6:**
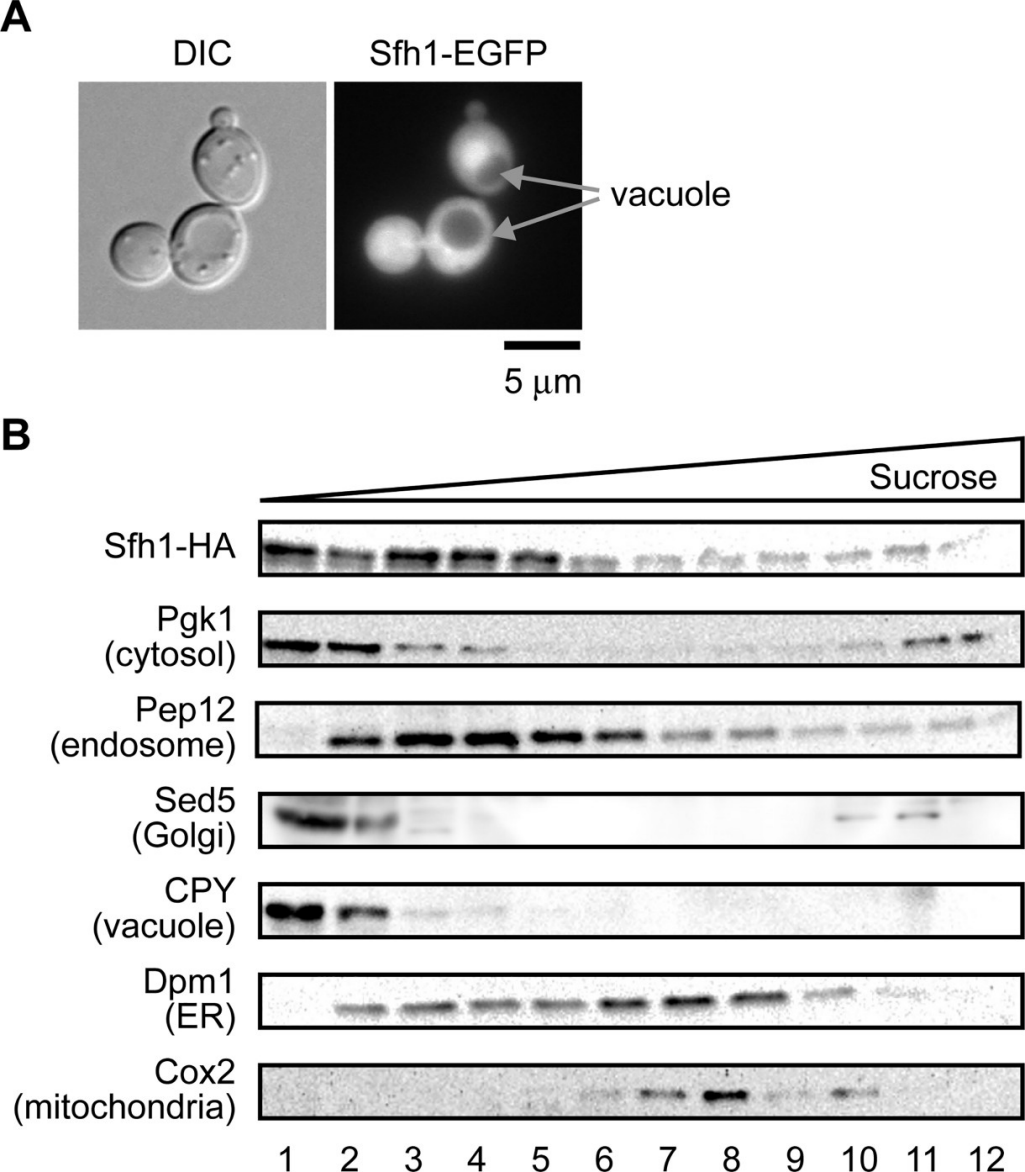
Sfh1 localizes in the cytosol, nucleus, and endosome, Golgi, and/or vacuole. (A) Sfh1-EGFP localizes in the cytosol and nucleus. Fluorescence microscopic observation of W303-1A cells overexpressing *SFH1-EGFP* cultured in SD medium to logarithmic phase. (B) Sfh1-HA localizes in the cytosol and endosome, Golgi, and/or vacuole. The cell extract of *psd1*Δ overexpressing *SFH1-HA* cultured in SD medium was fractionated by sucrose density gradient centrifugation. Distributions of Sfh1-HA and organelle marker proteins were evaluated by immunoblot.

The subcellular localization of the Sec14 family proteins has been studied by fluorescent microscopy and it was suggested that the Sfh1 fused with enhanced green fluorescent protein (Sfh1-EGFP) localizes to the cytosol and nucleus [57] and we obtained similar results (Fig 6A). However, fractionation by differential centrifugation suggested that Sfh1-EGFP localizes to the 30,000, 40,000, and 100,000 × g microsomal fractions, in addition to the cytosolic fraction [57]. To further assess the organelle to which Sfh1 localizes, we investigated the subcellular localization of Sfh1 tagged with the 3xHA epitope at its C-terminus (Sfh1-HA) by sucrose density gradient centrifugation. Analysis of the distribution of Sfh1-HA and organelle marker proteins suggested that, in addition to the cytosolic fraction, Sfh1-HA was enriched in the fractions containing the marker protein of the endosome, Golgi, and vacuole when overexpressed in the *psd1*Δ cells (Fig 6B and S7 Fig). Less Sfh1-HA was detected in the fractions, in which the ER and mitochondrial markers were enriched. Not only when overexpressed using a multi-copy vector, Sfh1-HA was also recovered in the fractions containing marker proteins of the endosome, Golgi, and vacuole when expressed under its native promoter using a low copy vector (S8 Fig). These results suggest that a fraction of Sfh1 localized to the endosome, Golgi, vacuole, and cytosol. As Psd2 has been reported to localize to the endosome, Golgi, and vacuole [9, 10], the localization of Sfh1-HA to those organelles could be important for enhancing Psd2-mediated PE synthesis.

### Deletion of *SFH1* impairs the growth of *psd1*Δ

**Fig 7:**
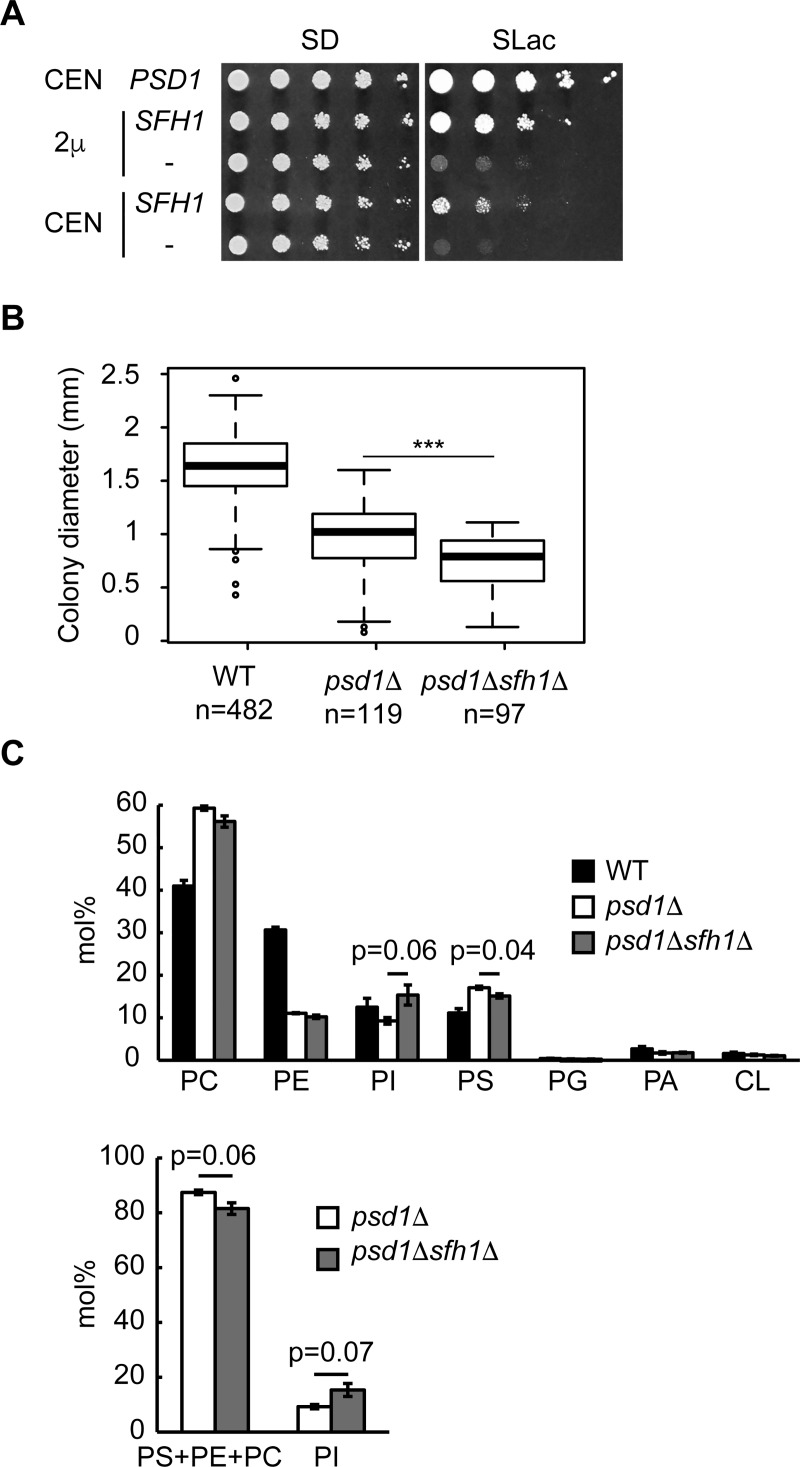
Deletion of *SFH1* impairs the growth of the *psd1*Δ strain on lactate. (A) Growth of *psd1*Δ expressing *SFH1*. Cells were cultured in SD medium to logarithmic phase and were spotted on SD or SLac medium in five-fold serial dilutions and were incubated on SD medium for 2 days or on SLac medium for 10 days. (B) Growth of *psd1*Δ*sfh1*Δ on lactate. Cells cultured in SD medium to logarithmic phase were harvested and suspended in distilled water. Cells of 1 x 10^−5^ OD_600_ unit were spread on SLac medium and incubated for 7 days. Colony diameter was measured and analyzed using Image J. *** represents p < 5e-8 by Man-Whitney U test. (C) Cellular phospholipid composition of *psd1*Δ and *psd1*Δ*sfh1*Δ cultured in SD medium. Lipids were extracted by the Bligh and Dyer’s method and were analyzed as described in the Material and Methods. Data are the means of three independent assays. Error bars represent S.E. p values of two-tailed Student’s t-test are shown.

Expression of *SFH1* using a low-copy vector also recovered the growth of *psd1*Δ, albeit to a lesser extent than overexpression of *SFH1* using a multi-copy vector (Fig 7A). *SFH1* has little effect on the growth of the wild-type *S*. *cerevisiae* strain [58], and its physiological role has remained obscure. To determine the physiological function of *SFH1*, we examined the growth of *psd1*Δ*sfh1*Δ. The growth of *psd1*Δ was slightly but significantly impaired by the deletion of *SFH1* both on solid and liquid SLac media (Fig 7B and S9 Fig).

To determine whether *SFH1* deletion affects phospholipid synthesis in *psd1*Δ, we analyzed the cellular phospholipid composition of *psd1*Δ*sfh1*Δ (Fig 7C). Compared to the *psd1*Δ cells, the proportion of PI tended to increase (*p* = 0.07) and the sum of PS, PE, and PC tended to decrease in the *psd1*Δ*sfh1*Δ cells (*p* = 0.06). These results imply that deletion of *SFH1* results in a slight depression in phospholipid synthesis through the PS branch of the CDP-DAG pathway in the *psd1*Δ cells.

### Involvement of MCSs in the suppression of *psd1*Δ by *SFH1* overexpression

Overexpression of *SFH1* increased the cellular and mitochondrial PE levels of *psd1*Δ in a Psd2-dependent manner, indicating that PE synthesized by Psd2 was supplied to the mitochondria. Contribution of MCSs in lipid transfer in cooperation with LTPs have been argued [19–21]. Mitochondria form MCSs with the ER by ERMES complex composed of Mmm1, Mdm10, Mdm12, and Mdm34 or the EMC composed of Emc1–Emc6. Mitochondria also form a MCS with vacuole (vCLAMP) marked by Vps39 and Ypt7, and a MCS with endosome by Vps13 in *S*. *cerevisiae* [22–26]. To determine whether these MCSs are required for the supplementation of PE synthesized by Psd2 to the mitochondria, *SFH1* was overexpressed in the *psd1*Δ strains, in which genes involved in these MCSs are simultaneously deleted, and their growth on lactate was observed.

**Fig 8:**
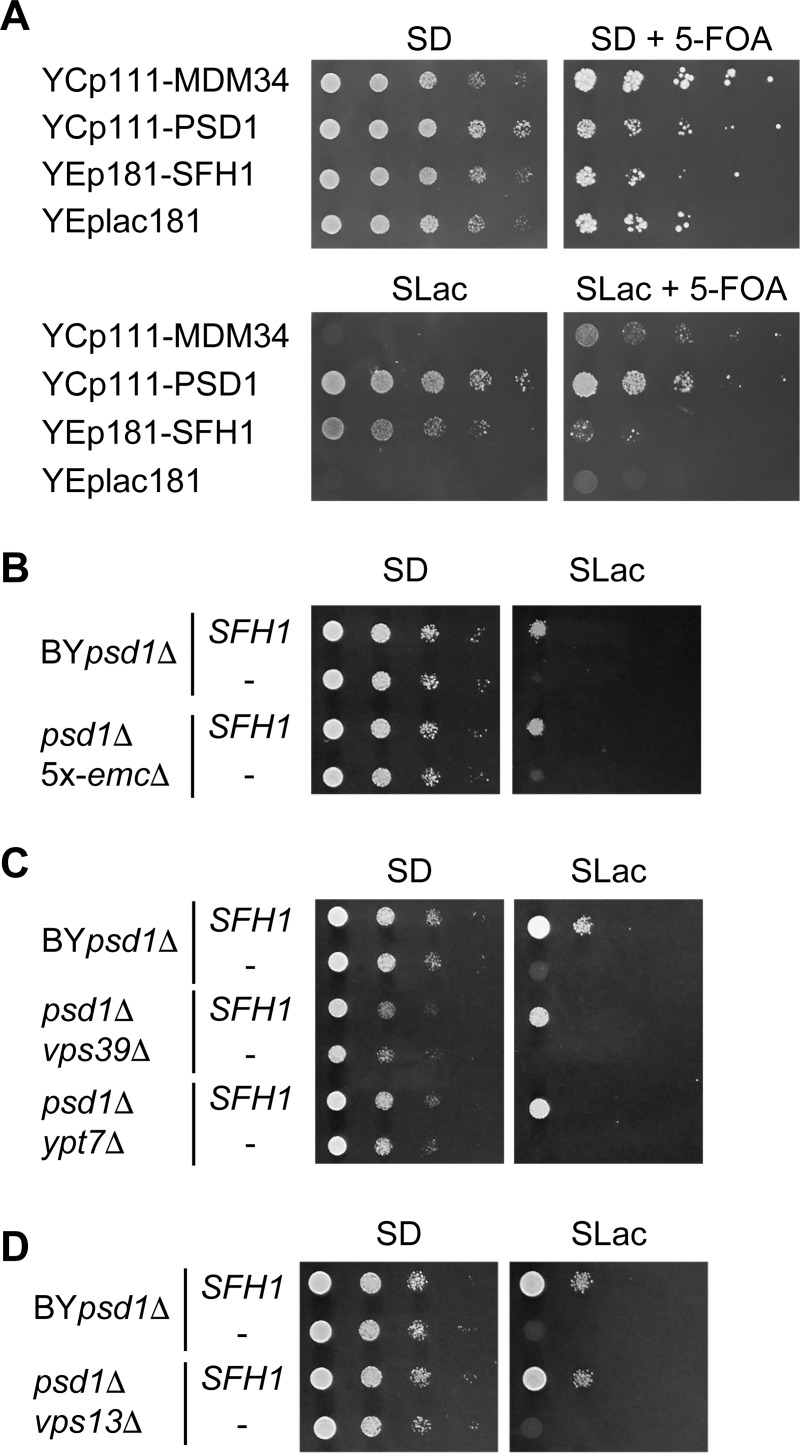
ERMES complex is important for recovery of growth of *psd1*Δ on lactate by *SFH1*. (A) *psd1*Δ*mdm34*Δ containing YCp33-MDM34 with YCp11-MDM34, YCp111-PSD1, YEp181-SFH1, or YEplac181 was cultured in SD medium to logarithmic phase and were spotted on SD or SLac medium with or without 0.5 mM 5-FOA in five-fold serial dilutions. Strains were incubated on SD or SLac medium without 5-FOA for 2 or 7 days, and on SD or SLac medium with 5-FOA for 7 or 10 days, respectively. Slight growth of *psd1*Δ*mdm34*Δ containing YCp11-MDM34 and YEplac181 on SLac medium in the presence of 5-FOA was probably due to longer incubation than that on SLac medium in the absence of 5-FOA. (B)–(D) Cells were cultured in SD medium to logarithmic phase and spotted on SD or SLac medium in ten-fold serial dilutions and were incubated on SD medium for 2 days or on SLac medium for 7 days.

Double deletion of *PSD1* and ERMES component genes exhibited much more severe growth defects on glucose or lactate in the presence or absence of Etn than the single deletion mutants, and the growth defect of *psd1*Δ*mdm34*Δ was not rescued by introduction of *PSD1* or *MDM34*, suggesting that the mitochondria were irreversibly damaged by the deletion of these genes (S10A Fig). Therefore, to examine the importance of the ERMES complex in the growth of *psd1*Δ supported by Sfh1 overproduction, we performed a plasmid shuffling assay for *MDM34* (Fig 8A). In this experiment, *MDM34* on the chromosome was deleted in *psd1*Δ harboring the plasmid containing *MDM34* with *URA3* as a marker (YCp33-MDM34). Then the low-copy plasmid carrying *MDM34* or *PSD1* or multi-copy plasmid carrying *SFH1* was introduced into the strain. These strains were cultured on SLac medium containing 5-FOA, which allows selection of cells that lost *URA3*, and growth of the cells that lost YCp33-MDM34 was observed. Overexpression of *SFH1* did not significantly affect the growth of *psd1*Δ*mdm34*Δ without the *MDM34*-harboring plasmid on SLac medium (Fig 8A, SLac + 5-FOA), suggesting that ERMES is critical for the growth of *psd1*Δ on lactate supported by Sfh1 overproduction. On the other hand, disruption of genes encoding the components of the EMC did not show a significant effect on growth of *psd1*Δ on lactate (Fig 8B and S10B Fig), implying that the EMC does not contribute to the increased supplementation of PE synthesized by Psd2 in the presence of *SFH1* overexpression to the mitochondria.

Deletion of *VPS39* impaired the growth of *psd1*Δ both on glucose and lactate (S10C Fig). Deletion of *YPT7* also slightly impaired the growth of *psd1*Δ (S10C Fig). The products of these two genes are also components of the HOPS complex involved in vacuole-vacuole or late endosome-vacuole tethering. Deletion of *VPS41*, a gene encoding a specific component of the HOPS complex, impaired the growth of *psd1*Δ to a similar degree to *VPS39* deletion (S10C Fig), and therefore the growth defects caused by *VPS39* or *YPT7* deletion may not be due to a defect in vCLAMP. Overexpression of *SFH1* restored the growth of both *psd1*Δ*vps39*Δ and *psd1*Δ*ypt7*Δ (Fig 8C), showing that vCLAMP is dispensable for the increased supply of PE synthesized by Psd2 in the presence of *SFH1* overexpression to the mitochondria. Deletion of *VPS13* did not exhibit a significant effect on the growth of *psd1*Δ (S10D Fig), and the growth restoration of *psd1*Δ*vps13*Δ on lactate by *SFH1* overexpression was comparable to that of *psd1*Δ (Fig 8D). These results suggested that *VPS13* is not required for the increased supplementation of PE synthesized by Psd2 in the presence of *SFH1* overexpression to the mitochondria in *psd1*Δ.

## Discussion

In this study, we identified *SFH1* as a multi-copy suppressor of the growth defect of *psd1*Δ on non-fermentable carbon sources and characterized its physiological function.

### Enhanced Psd2-mediated PE synthesis by Sfh1 overproduction

Overexpression of *SFH1* partially restored cellular and mitochondrial PE levels (Fig 3). One possible reason for this increase in the cellular PE level is the upregulation of the amounts or activities of Psd2 and Pss1, but we did not observe any significant change in protein abundance or activities of Psd2 and Pss1 following *SFH1* overexpression (Fig 4). Alternatively, Sfh1 could potentially activate Psd2 by regulating PIP signaling, as Sec14-family proteins are proposed to facilitate PI4P production by presenting PI to PI kinases [36, 37]. However, this possibility appears unlikely for the following reasons: i) Sfh1 does not activate the PI kinase *in vivo* [34], and ii) overexpression of the Sfh1 mutant deficient in PI binding partially but significantly suppressed the growth defect of *psd1*Δ on lactate (Fig 2C). In addition, activation of Pss1 or Psd2 by Sfh1 was not observed *in vitro* (Fig 4A and 4B). Activation of Pss1 appears to be harmful for *psd1*Δ mitochondria as overexpression of *PSS1* impaired the growth of *psd1*Δ particularly on lactate (Fig 5E), whereas the simultaneous overexpression of *SFH1* rescued the growth of *psd1*Δ (Fig 5E), implying that Sfh1 does not activate Pss1 solely *in vivo*.

**Fig 9:**
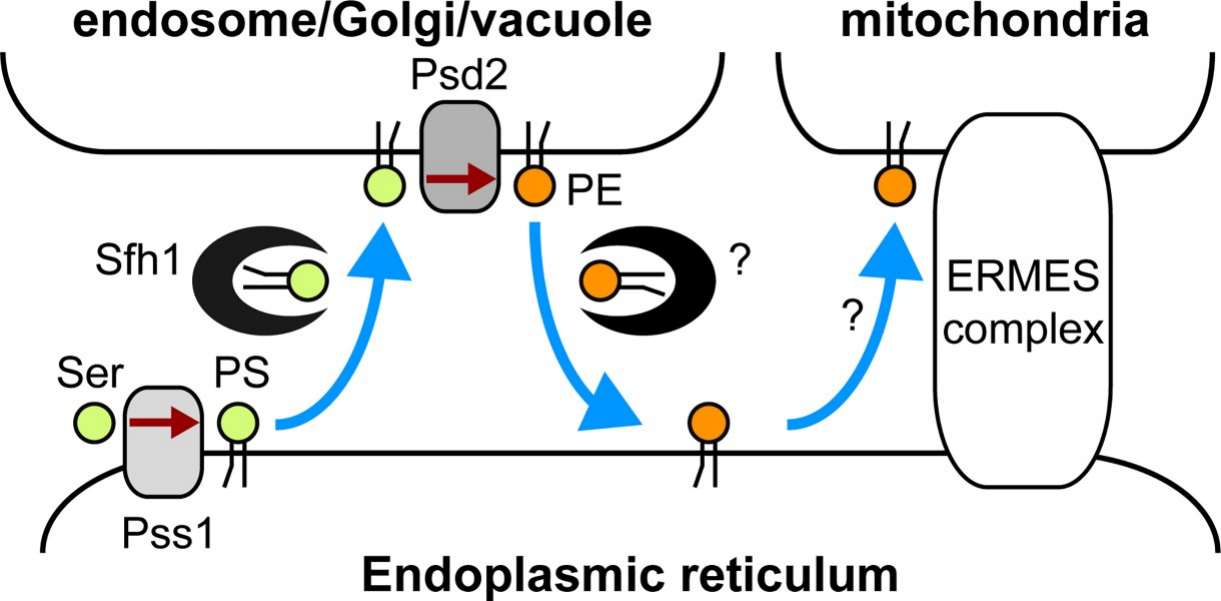
A model of phospholipid transport by Sfh1. Sfh1 may enhance transfer of PS synthesized in the ER to the endosome, Golgi, and vacuole, where PS is decarboxylated to PE by Psd2. Sfh1 may shuttle PE back to the ER, relieving the product inhibition of Psd2 by PE. PE is transferred from the ER to the mitochondria, possibly by ERMES complex.

The binding of Sfh1 to PS *in vivo* (Fig 5A), the possibility of the transport of NBD-labeled PS by Sfh1 *in vitro* (Fig 5C and 5D), and the localization of Sfh1 to the endosome, Golgi, and vacuole (Fig 6B) suggest that Sfh1 transports PS from the ER to those organelles and that PE synthesis is enhanced by the increased PS supply to Psd2 following Sfh1 overproduction (Fig 9). PS could be transported from the ER to endosome, Golgi, and vacuole through vesicle transport, but it might be insufficient. In addition, given that Sfh1 also bound to PE *in vivo* (Fig 5A) and *in vitro* [48], it is possible that PE synthesis increases because of the efficient export of PE from the endosome, Golgi, and vacuole to the ER or other organelles (Fig 9). Friedman *et al*. have reported that Psd1 is present in mitochondria and the ER, and indicate the importance of the optimal PE level to the ER function [11]. It may be possible that Sfh1 transfers PE synthesized by Psd2 to the ER to maintain an appropriate level of PE in the ER.

### Contribution of MCSs in the growth of *psd1*Δ overproducing Sfh1

A variety of MCSs have been discovered and their involvement in lipid transport has been discussed. One of the most well studied MCS components is the ERMES complex, which tethers the ER and mitochondria. While it has been reported that the ERMES components are dispensable for the transport of PS from the ER to mitochondria [59, 60], there is evidence that ERMES is involved in phospholipid transfer between the ER and mitochondria [22, 61–63]. In addition, ERMES has been shown to influence the mitochondrial phospholipid composition in concert with vCLAMP components or EMC [23, 24]. One possible reason for the requirement of ERMES for the growth suppression of *psd1*Δ on lactate by *SFH1* overexpression (Fig 8) is that PE synthesized by Psd2 is transported to the ER and then transported to mitochondria by an ERMES-dependent mechanism (Fig 9). Alternatively, the simultaneous deletion of the ERMES component genes with *PSD1* could have a deleterious effect on mitochondrial function, which cannot be recovered by Sfh1-dependent PE supplementation.

vCLAMP and the ER-endosome MCS formed by Vps13 were reported to dissociate from the mitochondria when cultured in medium containing the non-fermentable carbon source, glycerol or acetate [25, 26]. These MCSs may not participate in phospholipid transport during respiratory growth. As overexpression of *SFH1* restored the growth of *vps39*Δ*psd1*Δ or *vps13*Δ*psd1*Δ on lactate (Fig 8), it is conceivable that PE synthesized by Psd2 was transported to the mitochondria through different routes. Disruption of EMC had little effect on the growth of *psd1*Δ. Lahiri *et al*. reported that 5x-*emc*, in which *EMC1*, *EMC2*, *EMC3*, *EMC5*, and *EMC6* are deleted, did not grow on the rich medium containing glycerol (YPGly) and had a growth defect on the rich medium containing glucose (YPD) [23]. However, the strain we constructed (5x-*emc*Δ) grew similarly to the wild-type strain on the minimal medium containing glucose or lactate (SD or SLac) (S10B Fig). This discrepancy may be due to the difference in medium and/or the genetic backgrounds. Although *VPS13* and *EMC* genes were dispensable for the growth recovery of *psd1*Δ by *SFH1* overexpression, we cannot exclude the possibility that the MCSs formed by these proteins partially contribute to the import of PE synthesized by Psd2 to the mitochondria. In addition, it is possible that PerMit contact is involved in phospholipid transport to mitochondria [29].

### Physiological role of Sfh1

Although Sfh1 exhibits the highest similarity to Sec14 in the *S*. *cerevisiae* Sec14 family of proteins, deletion of *SFH1* in the wild-type strain does not confer a significant effect on growth. Other Sec14 family members have been reported to be involved in various cellular processes [33, 64–67], yet the physiological role of Sfh1 has remained unclear. In this study, it was suggested that Sfh1 has a physiological function distinct from other Sfh proteins in *S*. *cerevisiae*. Deletion of *SFH1* decreased the sum of the levels of PS, PE, and PC and increased PI level in *psd1*Δ cells (Fig 7C). This could be explained by our suggestion that Sfh1 is involved in the phospholipid transport between the ER and endosome, Golgi, and/or vacuole and less effective phospholipid transport between these organelles by *SFH1* deletion partially abrogated the PS branch of the CDP-DAG pathway, resulting in increased conversion of the excess of CDP-DAG to PI.

*SFH1* deletion aggravated the growth of *psd1*Δ on SLac albeit to a small extent (Fig 7B and S9 Fig). *SEC14*, and perhaps other *SFH* genes, may function redundantly with *SFH1*, reducing the impact of *SFH1* disruption. *SFH1* and *SEC14* are supposed to be generated by whole genome duplication [68], and these two genes may partially share functions. If this is the case, then where does the functional difference arise from? Sec14 was reported to localize to the cytosol and Golgi, while Sfh1 was suggested to localize to the cytosol, nucleus, endosome, Golgi, and vacuole [57, 69] (Fig 6). This difference in the subcellular localization may affect their functions. Moreover, Sec14 has high PITP/PCTP activities whereas those of Sfh1 are very low. High PITP/PCTP activities of Sec14 may be derived from strong binding preferences for PI and PC over PS and PE.

*SFH4* has been reported to play a critical role in PE synthesis by Psd2 [70]. Nonetheless, overexpression of *SFH4* did not rescue the growth defect of *psd1*Δ on lactate (Fig 2B). As Sfh4 forms a complex with Psd2 and Pbi1, overexpression of *SFH4* alone may not influence the rate of PE production by Psd2. Overexpression of *SFH4* partially rescued the growth defect of *sec14*^ts^ at a restrictive temperature, indicating that there is some functional redundancy between *SEC14* and *SFH4* [57]. In contrast, *SFH1* appears to have a distinct physiological function from *SFH4* (Fig 2B). Indeed, while Sfh1 was suggested to transfer NBD-PS *in vitro* (Fig 5C and 5D), Sfh4 has been reported not to possess *in vitro* PS transfer activity [67].

Large-scale surveys have revealed that the expression of *SFH1* is induced under various stress conditions, including high osmolality, high salt concentrations, temperature shifts, rapamycin treatment, and the diauxic shift [71–73]. Vacuole is involved in the responses to high osmolality, high salt concentrations, and rapamycin treatment [74]. PE synthesized by Psd2 was suggested to be important for vacuolar function [10], and PE synthesis by Psd2 could be upregulated in response to these stresses by transcriptional induction of *SFH1*. During the diauxic shift, yeast cells adapt themselves to respiring growth by the induction of genes involved in the assimilation of non-fermentable carbon sources and proliferation of the mitochondria. Enhanced expression of *SFH1* during the diauxic shift implies that Sfh1 participates in mitochondrial function. Further study is required to clarify the physiological role of Sfh1 under these stress conditions.

## Supporting information

S1 Fig Overexpression of *SFH1* restores the growth of the *psd1*Δ strain on non-fermentable carbon sources. (A) Overexpression of *SFH1* restores the growth of *psd1*Δ on various non-fermentable carbon sources. Strains were spotted on synthetic medium with glucose, glycerol, ethanol, and lactate as sole carbon sources in ten-fold serial dilutions and were incubated on glucose for 2 days or on glycerol, ethanol, and lactate for 7 days. (B) *SFH4* is critical to recover the growth of *psd1*Δ on lactate by *SFH1* overexpression. Strains were spotted on SD or SLac medium in the presence or absence of 1 mM Etn in ten-fold serial dilutions and were incubated for 2 or 7 days, respectively. (C) Simultaneous overexpression of *SFH1* and *PSD2* leads to improved growth of *psd1*Δ on lactate compared to single overexpression of one of these genes. Strains were spotted on SD or SLac medium in five-fold serial dilutions and were incubated for 2 or 7 days, respectively.
(TIF)

S2 Fig Expression of Sfh1 mutants. (A) The *psd1*Δ strains overexpressing *SFH1*, *SFH1-EGFP*, *sfh1*^*S175I*,*T177I*^, *sfh1*^*S175I*,*T177I*^*-EGFP*, *sfh1*^*R61A*,*T238D*^, *sfh1*^*R61A*,*T238D*^*-EGFP*, *sfh1*^*L179W*,*I196W*^, and *sfh1*^*L179W*,*I196W*^*-EGFP* were cultured in SD medium to logarithmic phase and were spotted on SD or SLac media in ten-fold serial dilutions and were incubated on SD medium for 2 days or on SLac medium for 7 days. (B) The *psd1*Δ strains overexpressing *SFH1*, *SFH1-EGFP*, *sfh1*^*S175I*,*T177I*^, *sfh1*^*S175I*,*T177I*^*-EGFP*, *sfh1*^*R61A*,*T238D*^, *sfh1*^*R61A*,*T238D*^*-EGFP*, *sfh1*^*L179W*,*I196W*^, and *sfh1*^*L179W*,*I196W*^*-EGFP* were cultured in SD medium to logarithmic phase and the levels Sfh1 proteins tagged with EGFP were evaluated by immunoblot using anti-EGFP antibody.
(TIF)

S3 Fig Overexpression of *SFH1* does not restore the cellular PE level in *psd1*Δ*psd2*Δ. Cellular phospholipid composition of *psd1*Δ*psd2*Δ overexpressing *SFH1* cultured in semi-synthetic lactate medium was determined. Data are the means of three independent assays. Error bars represent S.E. n.s., not significant.
(TIF)

S4 Fig Overexpression of *SFH1* did not increase the protein levels of Pss1 and Psd2. Uncropped blots of Fig 4C and 4D in triplicate are shown.
(TIF)

S5 Fig Binding of Sfh1 with phospholipids *in vivo* and phospholipid transfer by Sfh1 *in vitro*. (A) Sfh1 fused with ZZ tag is functional in *S*. *cerevisiae*. Strains were spotted on SD or SLac medium in ten-fold serial dilutions and were incubated for 2 or 7 days, respectively. (B) Affinity purification of Sfh1-ZZ and Sfh1^S175I,T177I^-ZZ. Affinity purified proteins were eluted from IgG sepharose beads and concentrated by TCA precipitation. The purity was checked by SDS-PAGE. Arrow head represents Sfh1-ZZ and Sfh1^S175I,T177I^-ZZ. * represents contaminated proteins. (C) NBD-PS dequenching assay was performed in the absence of acceptor liposomes. (D) and (E) NBD-PC (D) and NBD-PE (E) transfer activities of Sfh1 and Sfh1^S175I,T177I^ mutant were measured at room temperature. NBD fluorescence intensities were set to 0 at 0 s. Data are the means of three independent assays. Error bars represent S.E. Proteins were added to final concentration of 800 nM. (F) Overexpression of *SFH4* does not rescue the growth of *psd1*Δ overexpressing *PSS1*. Strains were spotted on SD or SLac medium in the presence or absence of 1 mM Etn in ten-fold serial dilutions and were incubated for 2 or 7 days, respectively.
(TIF)

S6 Fig Cellular phospholipid compositions of the *psd1*Δ strains overexpressing *PSS1* and *SFH1*. The *psd1*Δ strains harboring YEplac181 and YEplac195, YEp181-PSS1 and YEplac195, and YEp181-PSS1 and YEp195-SFH1 were cultured in SD medium to late logarithmic phase. Lipids were extracted and analyzed. Data are the means of three independent assays.
(TIF)

S7 Fig Sfh1-HA localizes in the cytosol and endosome, Golgi, and/or vacuole. Uncropped blots of Fig 6B are shown.
(TIF)

S8 Fig Sfh1-HA localizes in the cytosol and endosome, Golgi, and/or vacuole. (Upper panels) Cell extract of *psd1*Δ expressing *SFH1-HA* by a low copy vector cultured in SD medium was fractionated by sucrose density gradient centrifugation. Distributions of Sfh1-HA and organelle marker proteins were evaluated by immunoblot. (Lower panels) Uncropped blots are shown.
(TIF)

S9 Fig Growth curve of *psd1*Δ*sfh1*Δ on SD and SLac without Etn supplementation. Growth of the wild-type strain (circles), *psd1*Δ (squares), and *psd1*Δ*sfh1*Δ (triangles) in SD (open symbols) or SLac (closed symbols) media was analyzed as described in Materials and methods.
(TIF)

S10 Fig Genetic interaction between *PSD1* and the genes encoding the components of the membrane contact sites. (A)–(D) Strains were spotted on SD or SLac media in the presence or absence of 1 mM Etn in ten-fold serial dilutions and were incubated for 2 or 7 days, respectively. (A) Disruption of ERMES component genes in *psd1*Δ leads to irreversible loss of mitochondrial function. (B) Disruption of EMC component genes does not affect the growth of *psd1*Δ. (C) Disruption of vCLAMP / HOPS complex genes aggravate the growth of *psd1*Δ. (D) Disruption of *VPS13* does not affect the growth of *psd1*Δ.
(TIF)

S1 Table Primers used in this study. (DOCX)
