# Supplementary figures and images for: Suppression of respiratory growth defect of mitochondrial phosphatidylserine decarboxylase deficient mutant by overproduction of Sfh1, a Sec14 homolog, in yeast
Source: PLoS One. 2019 Apr 8;14(4):e0215009. doi: 10.1371/journal.pone.0215009 (PMC6453485; doi:10.1371/journal.pone.0215009)

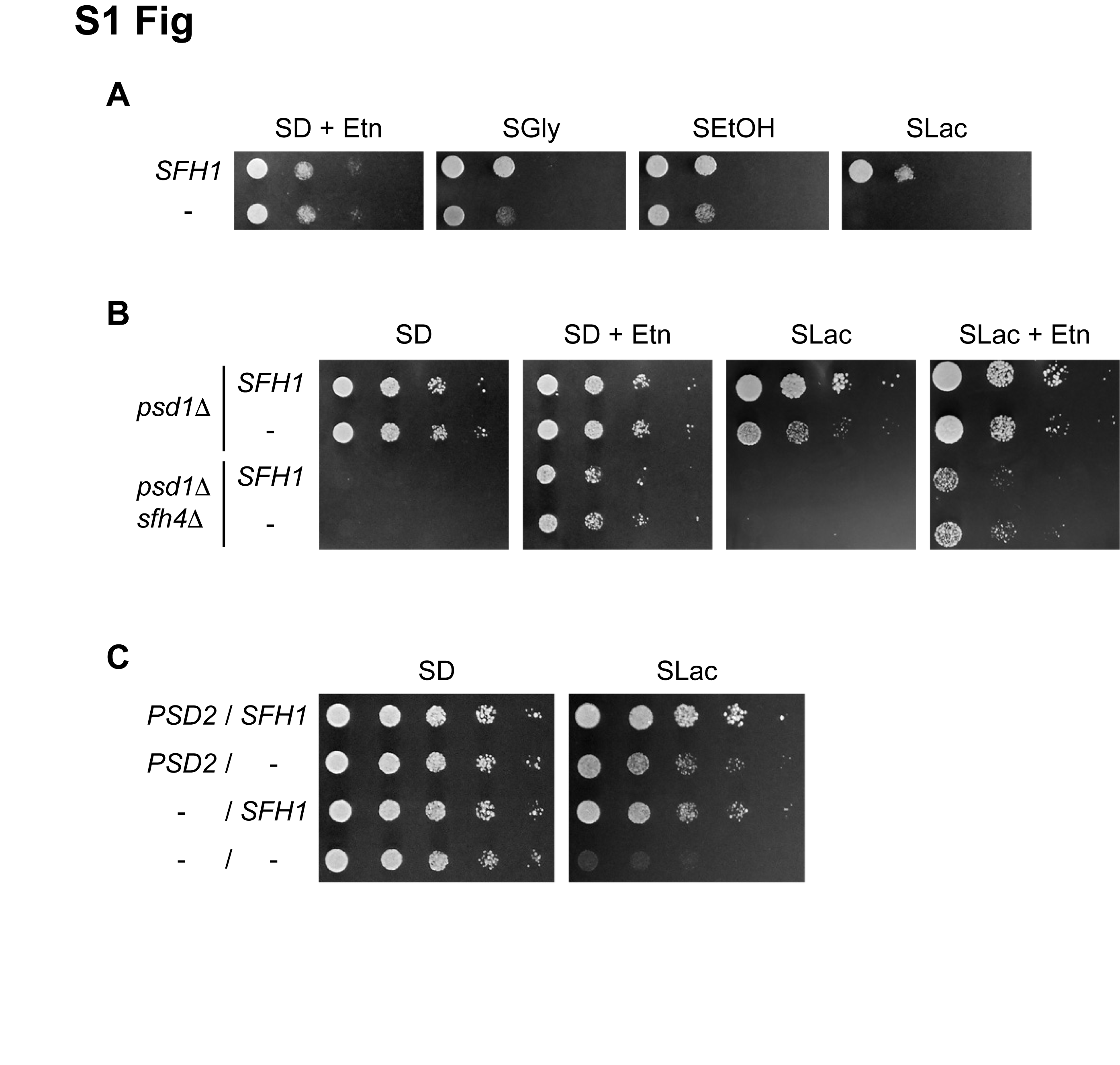

Supplement: S1 Fig — (A) Overexpression of SFH1 restores the growth of psd1Δ on various non-fermentable carbon sources. Strains were spotted on synthetic medium with glucose, glycerol, ethanol, and lactate as sole carbon sources in ten-fold serial dilutions and were incubated on glucose for 2 days or on glycerol, ethanol, and lactate for 7 days. (B) SFH4 is critical to recover the growth of psd1Δ on lactate by SFH1 overexpression. Strains were spotted on SD or SLac medium in the presence or absence of 1 mM Etn in ten-fold serial dilutions and were incubated for 2 or 7 days, respectively. (C) Simultaneous overexpression of SFH1 and PSD2 leads to improved growth of psd1Δ on lactate compared to single overexpression of one of these genes. Strains were spotted on SD or SLac medium in five-fold serial dilutions and were incubated for 2 or 7 days, respectively. (TIF) [file pone.0215009.s001.tif]

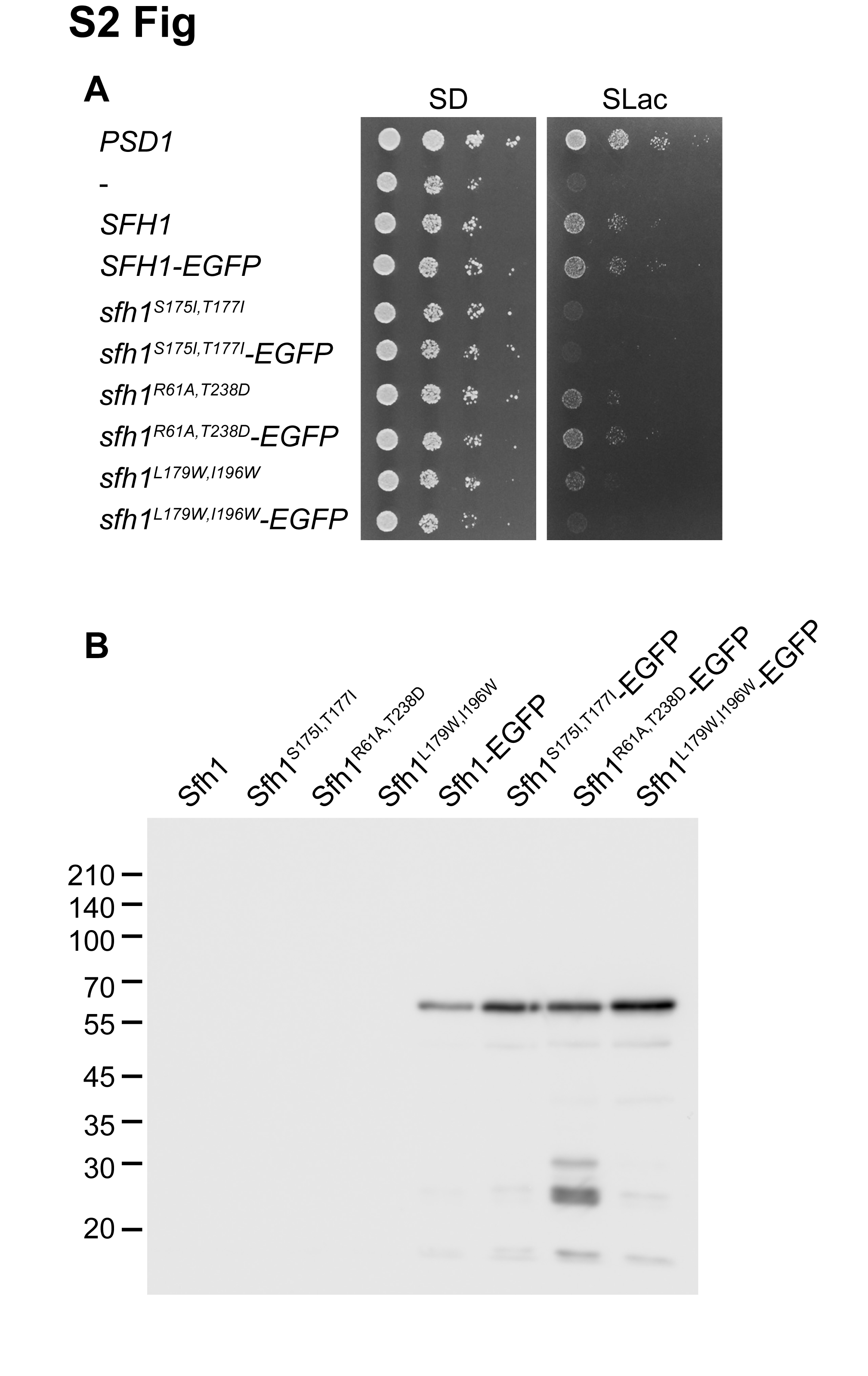

Supplement: S2 Fig — (A) The psd1Δ strains overexpressing SFH1, SFH1-EGFP, sfh1S175I,T177I, sfh1S175I,T177I-EGFP, sfh1R61A,T238D, sfh1R61A,T238D-EGFP, sfh1L179W,I196W, and sfh1L179W,I196W-EGFP were cultured in SD medium to logarithmic phase and were spotted on SD or SLac media in ten-fold serial dilutions and were incubated on SD medium for 2 days or on SLac medium for 7 days. (B) The psd1Δ strains overexpressing SFH1, SFH1-EGFP, sfh1S175I,T177I, sfh1S175I,T177I-EGFP, sfh1R61A,T238D, sfh1R61A,T238D-EGFP, sfh1L179W,I196W, and sfh1L179W,I196W-EGFP were cultured in SD medium to logarithmic phase and the levels Sfh1 proteins tagged with EGFP were evaluated by immunoblot using anti-EGFP antibody. (TIF) [file pone.0215009.s002.tif]

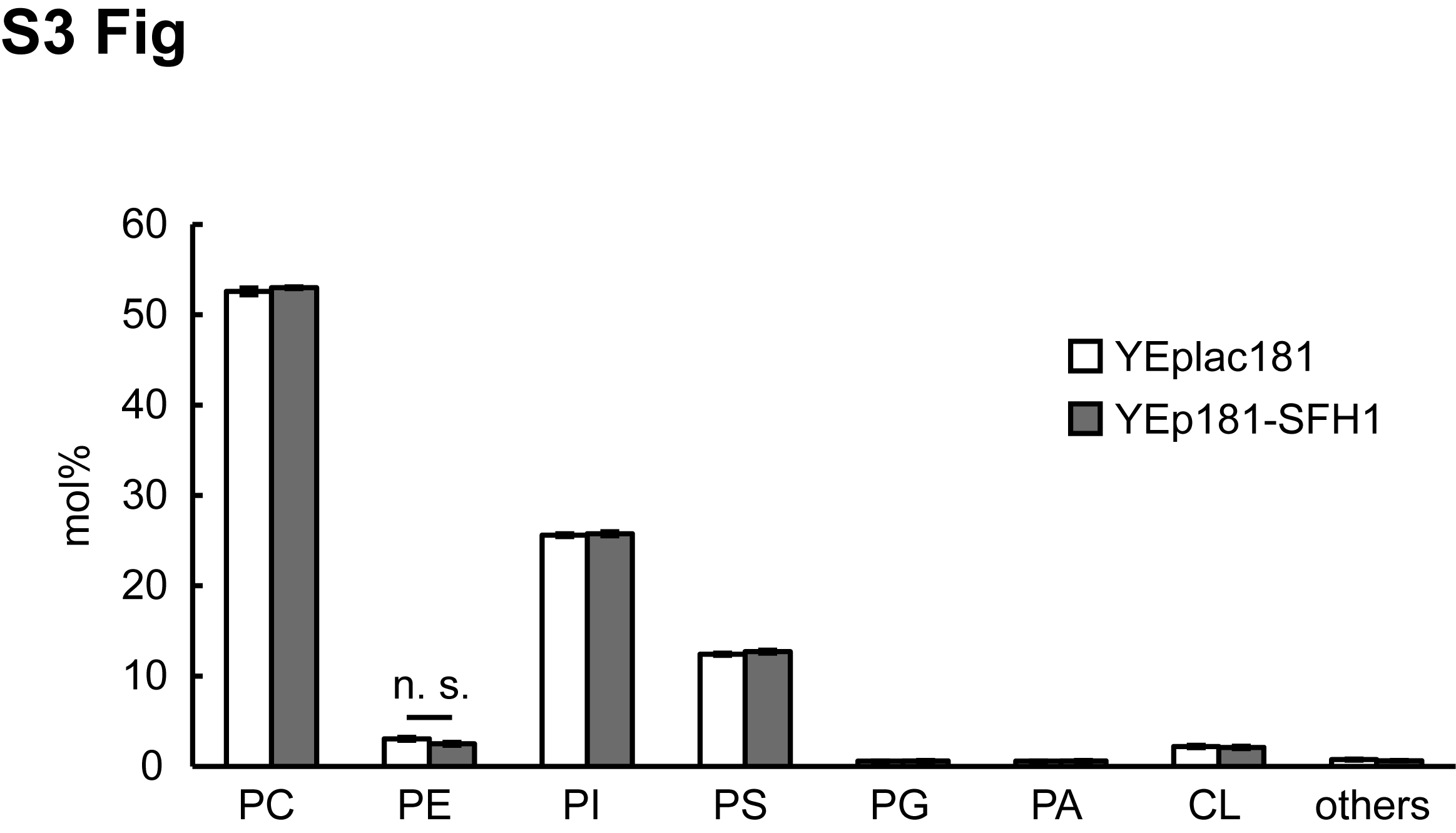

Supplement: S3 Fig — Cellular phospholipid composition of psd1Δpsd2Δ overexpressing SFH1 cultured in semi-synthetic lactate medium was determined. Data are the means of three independent assays. Error bars represent S.E. n.s., not significant. (TIF) [file pone.0215009.s003.tif]

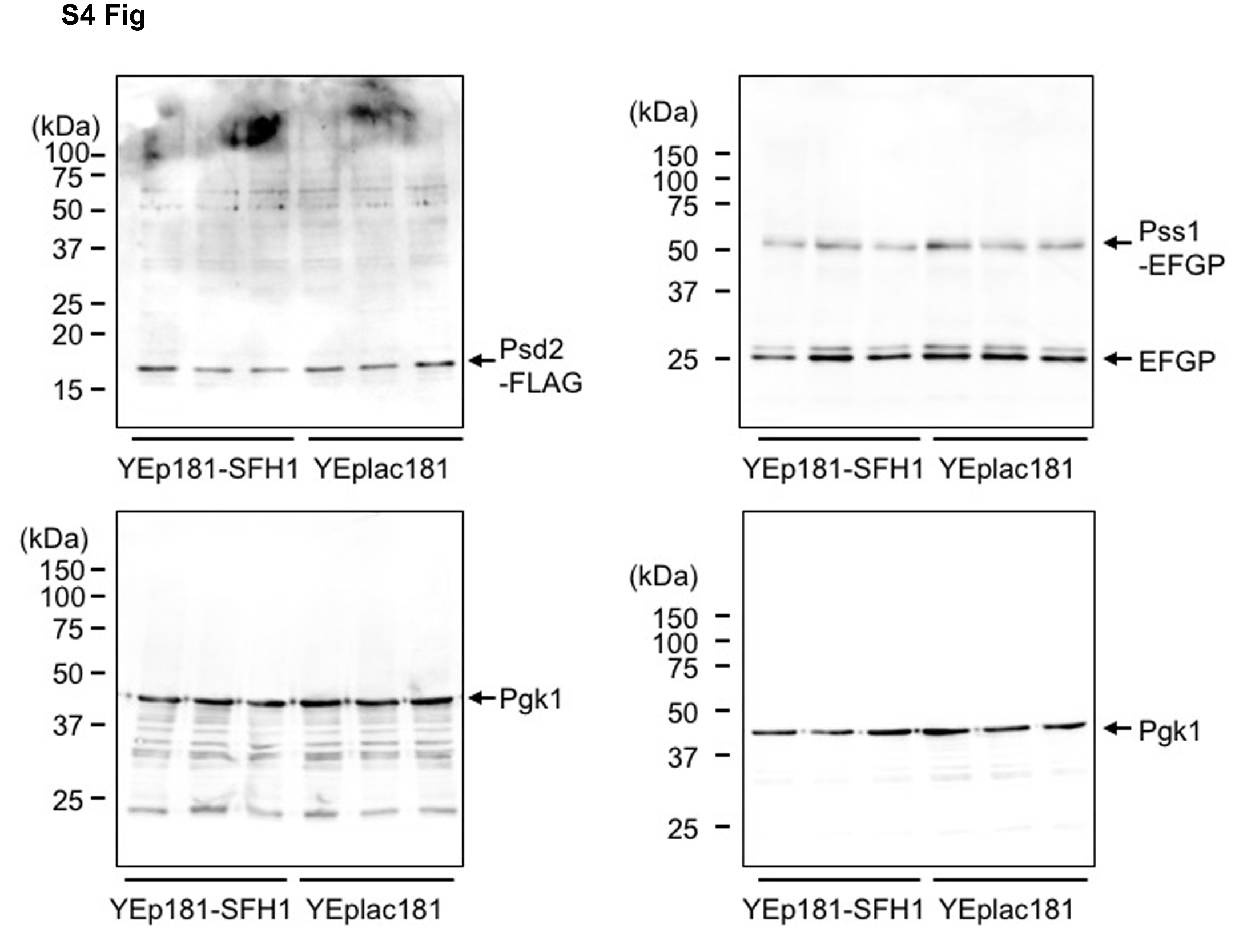

Supplement: S4 Fig — Uncropped blots of Fig 4C and 4D in triplicate are shown. (TIF) [file pone.0215009.s004.tif]

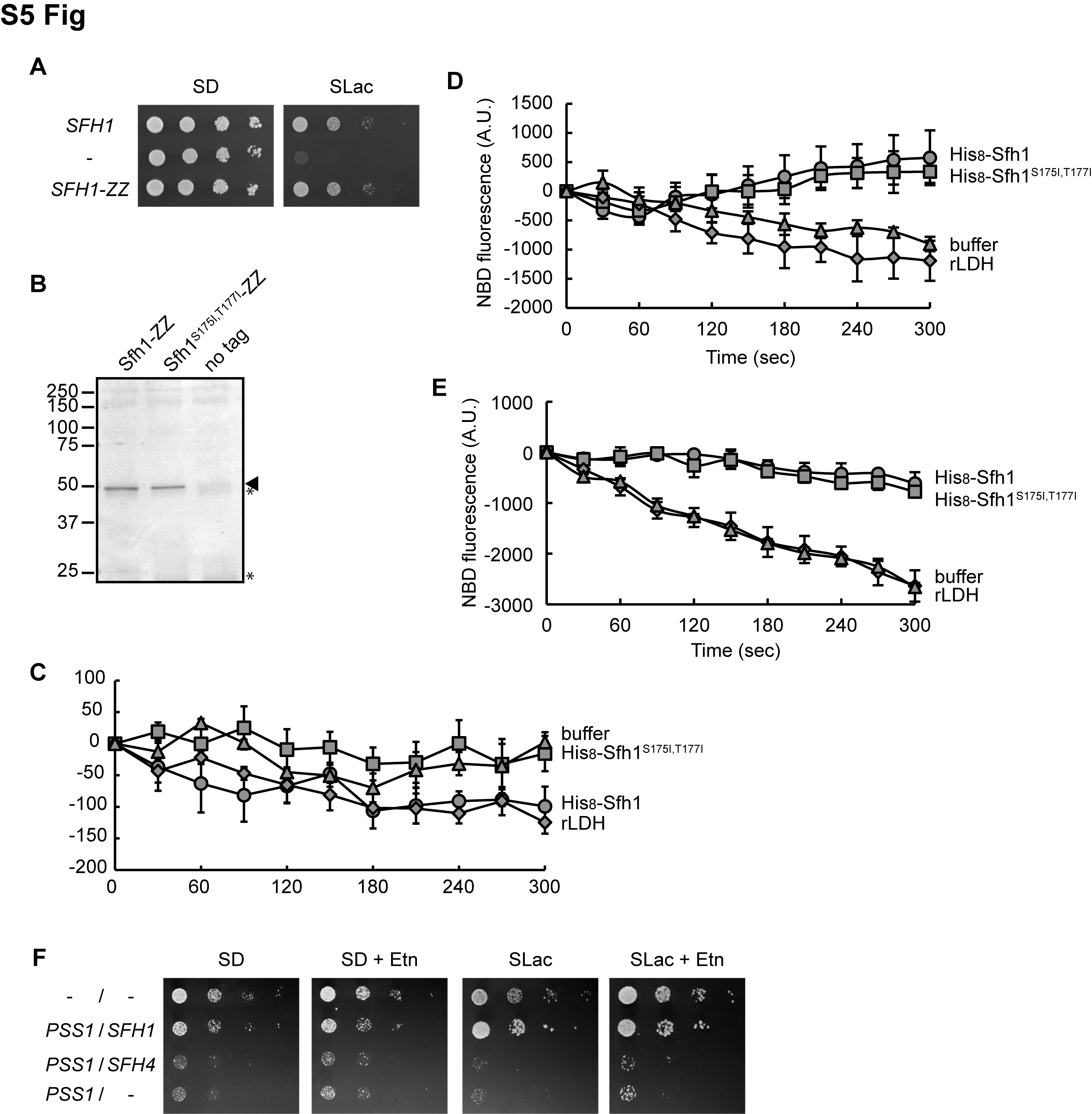

Supplement: S5 Fig — (A) Sfh1 fused with ZZ tag is functional in S. cerevisiae. Strains were spotted on SD or SLac medium in ten-fold serial dilutions and were incubated for 2 or 7 days, respectively. (B) Affinity purification of Sfh1-ZZ and Sfh1S175I,T177I-ZZ. Affinity purified proteins were eluted from IgG sepharose beads and concentrated by TCA precipitation. The purity was checked by SDS-PAGE. Arrow head represents Sfh1-ZZ and Sfh1S175I,T177I-ZZ. * represents contaminated proteins. (C) NBD-PS dequenching assay was performed in the absence of acceptor liposomes. (D) and (E) NBD-PC (D) and NBD-PE (E) transfer activities of Sfh1 and Sfh1S175I,T177I mutant were measured at room temperature. NBD fluorescence intensities were set to 0 at 0 s. Data are the means of three independent assays. Error bars represent S.E. Proteins were added to final concentration of 800 nM. (F) Overexpression of SFH4 does not rescue the growth of psd1Δ overexpressing PSS1. Strains were spotted on SD or SLac medium in the presence or absence of 1 mM Etn in ten-fold serial dilutions and were incubated for 2 or 7 days, respectively. (TIF) [file pone.0215009.s005.tif]

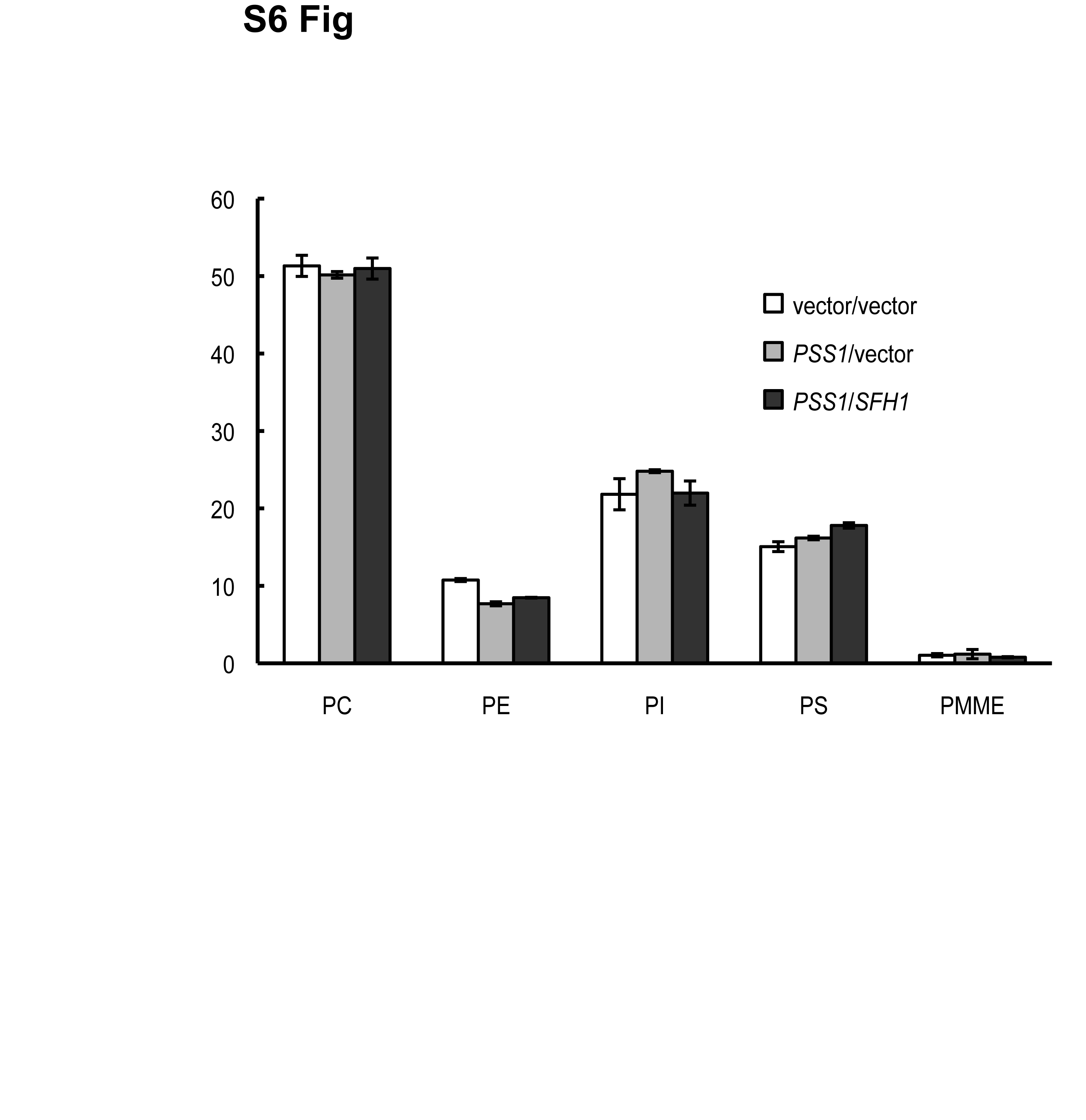

Supplement: S6 Fig — The psd1Δ strains harboring YEplac181 and YEplac195, YEp181-PSS1 and YEplac195, and YEp181-PSS1 and YEp195-SFH1 were cultured in SD medium to late logarithmic phase. Lipids were extracted and analyzed. Data are the means of three independent assays. (TIF) [file pone.0215009.s006.tif]

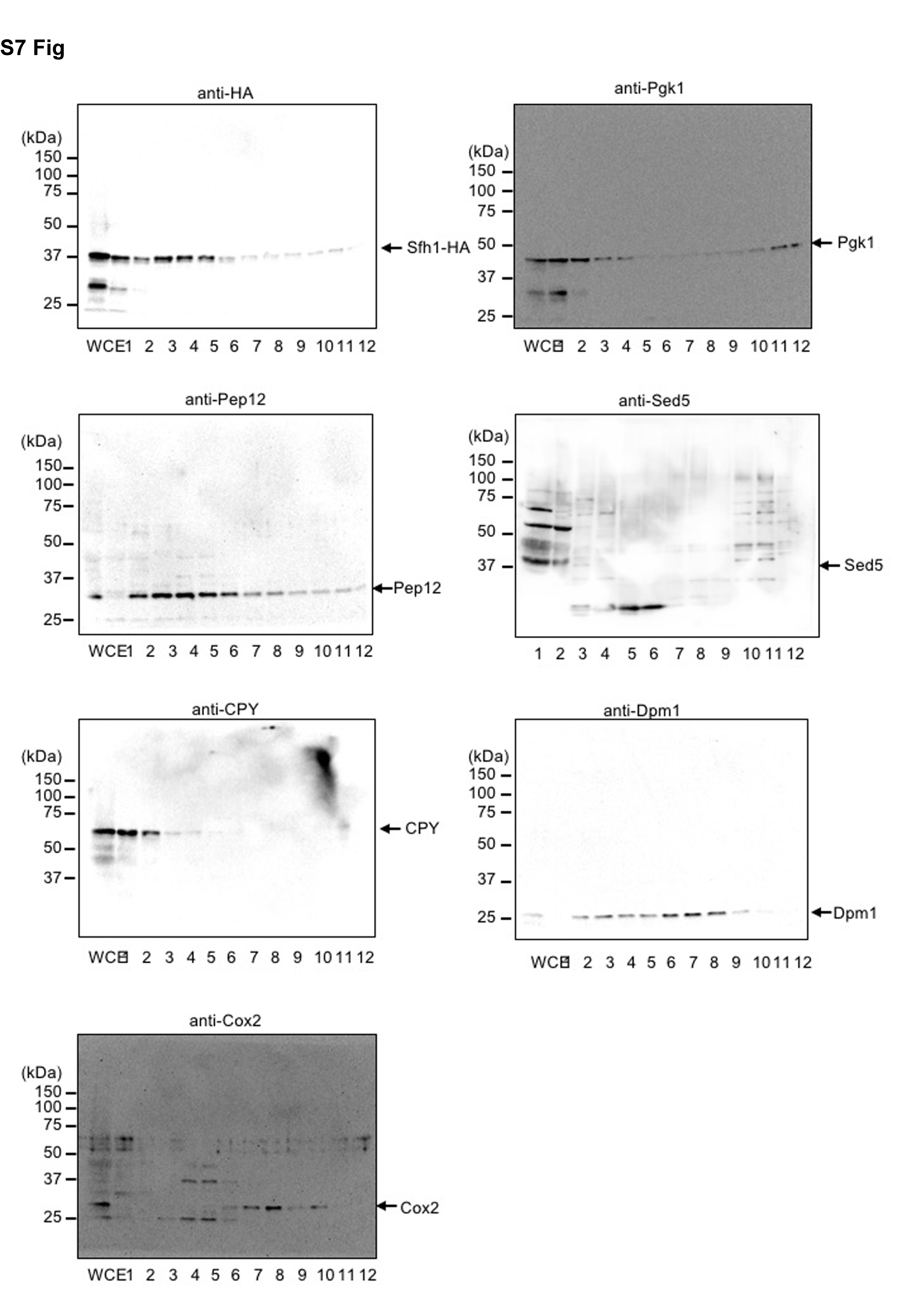

Supplement: S7 Fig — Uncropped blots of Fig 6B are shown. (TIF) [file pone.0215009.s007.tif]

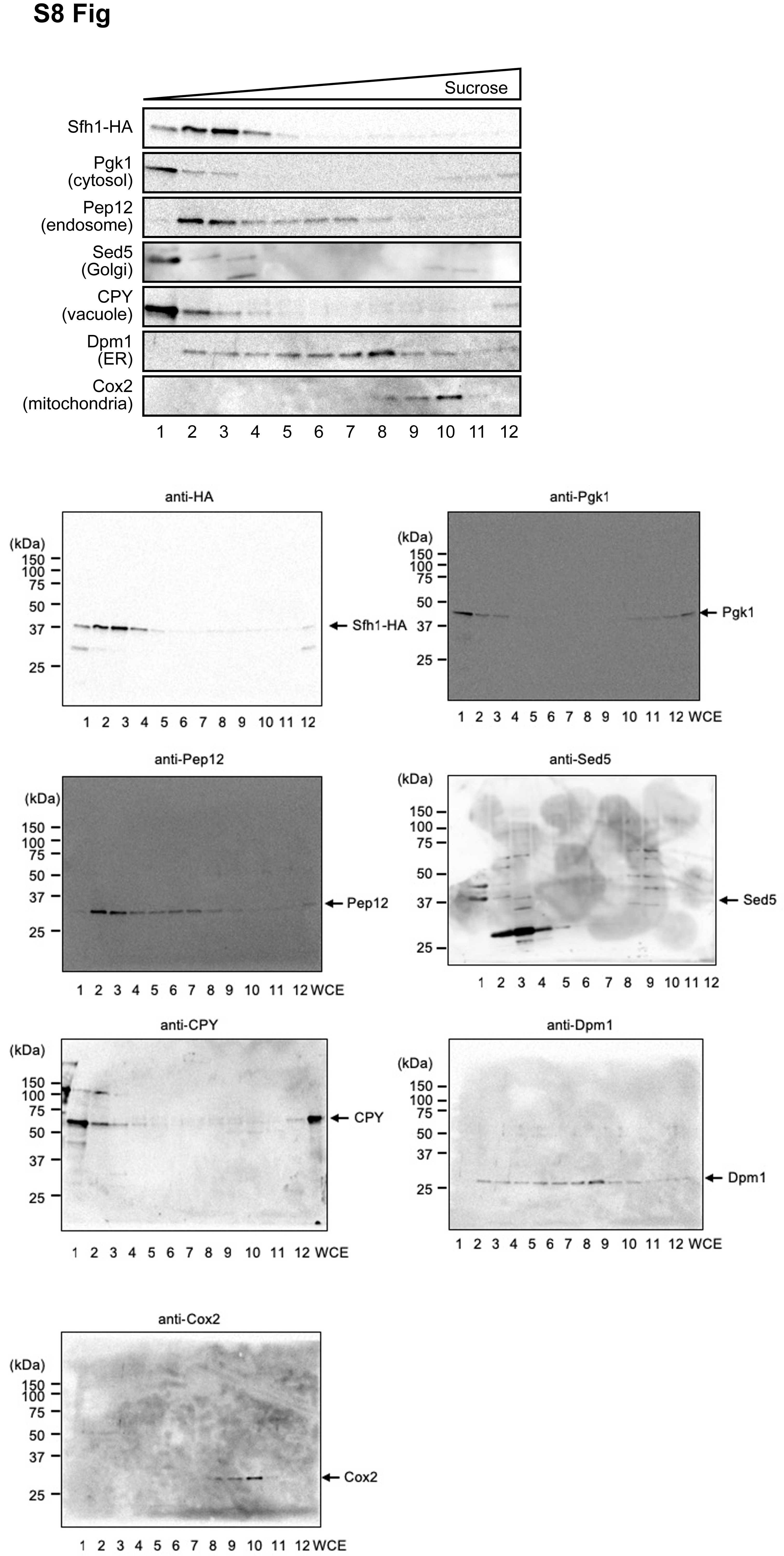

Supplement: S8 Fig — (Upper panels) Cell extract of psd1Δ expressing SFH1-HA by a low copy vector cultured in SD medium was fractionated by sucrose density gradient centrifugation. Distributions of Sfh1-HA and organelle marker proteins were evaluated by immunoblot. (Lower panels) Uncropped blots are shown. (TIF) [file pone.0215009.s008.tif]

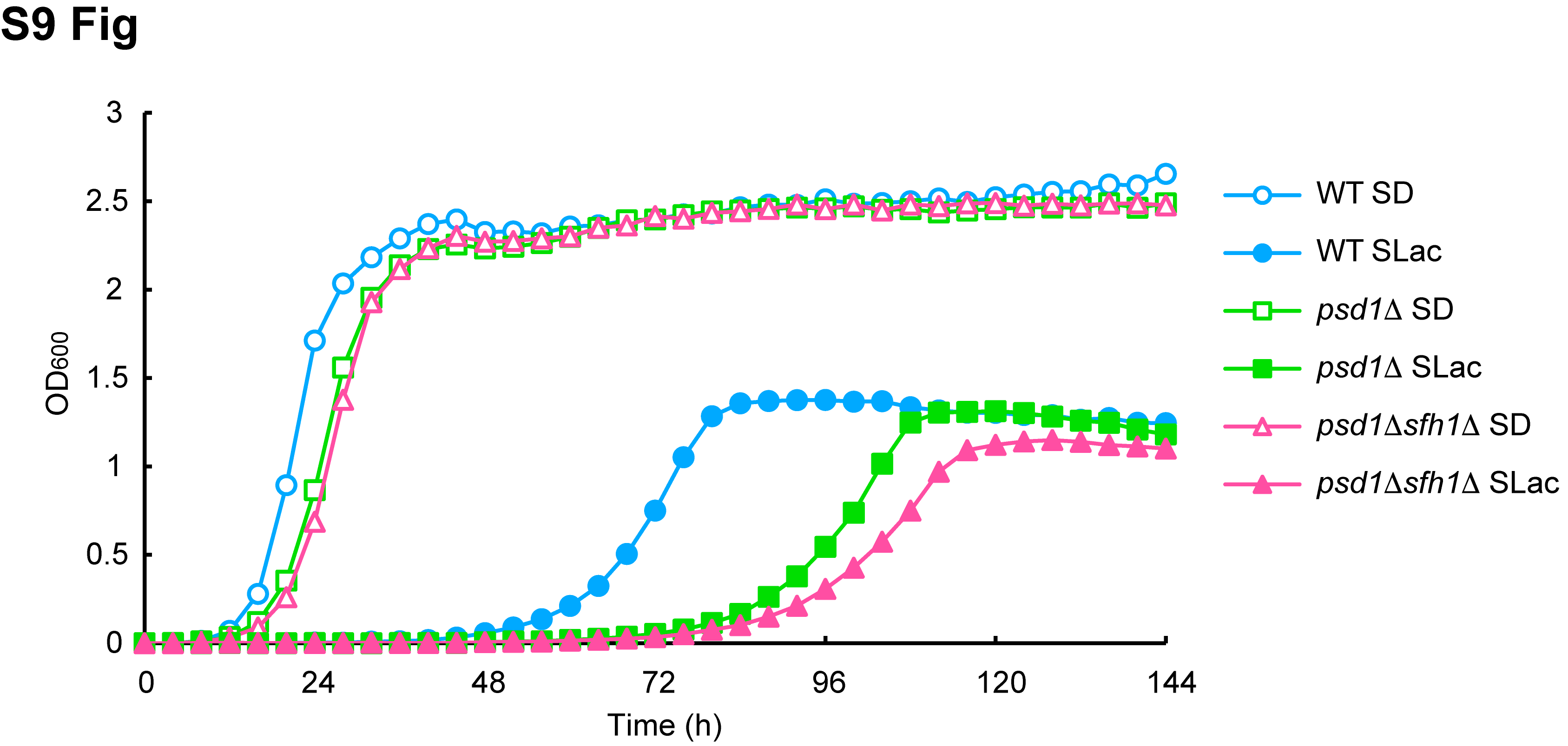

Supplement: S9 Fig — Growth of the wild-type strain (circles), psd1Δ (squares), and psd1Δsfh1Δ (triangles) in SD (open symbols) or SLac (closed symbols) media was analyzed as described in Materials and methods. (TIF) [file pone.0215009.s009.tif]

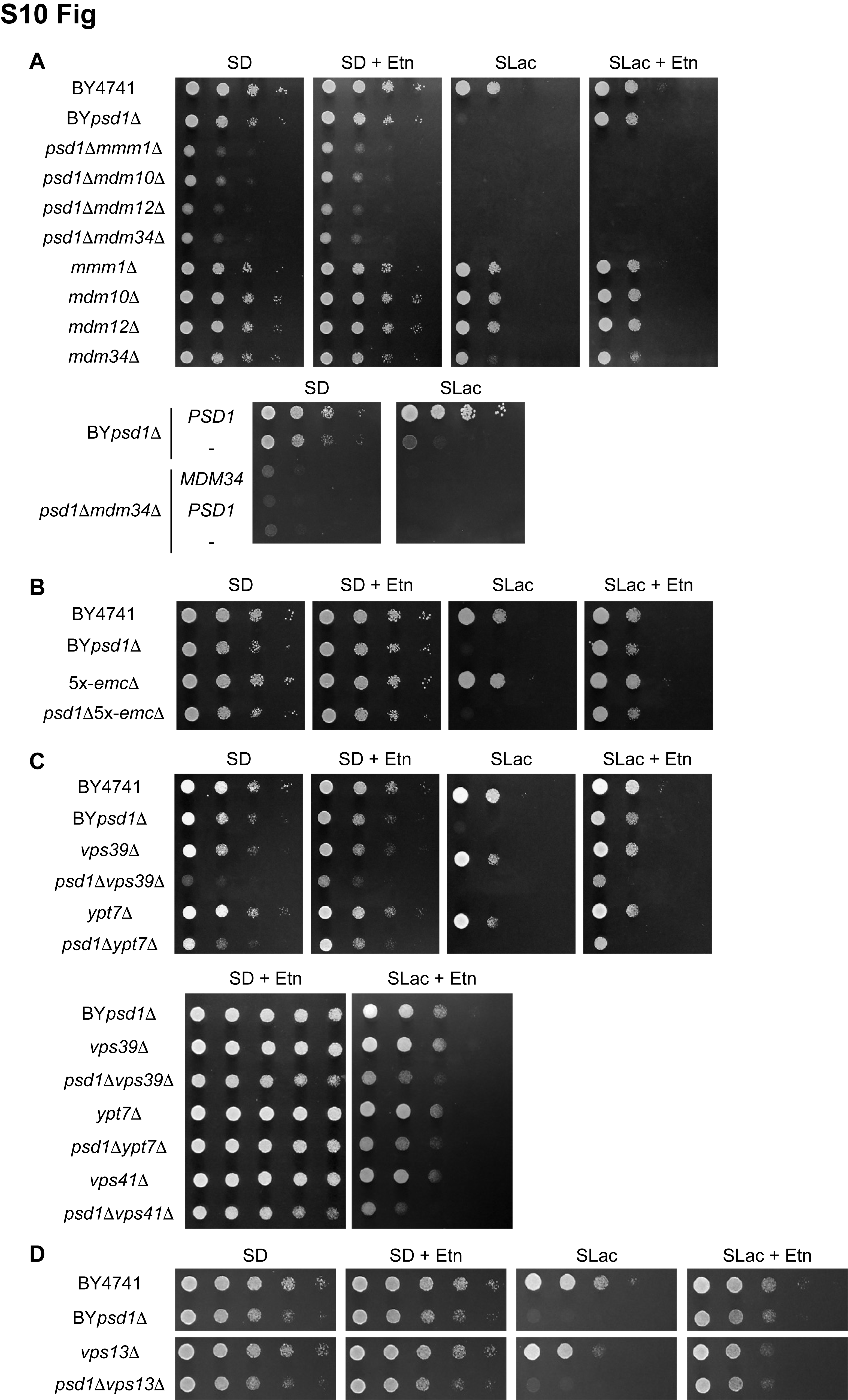

Supplement: S10 Fig — (A)–(D) Strains were spotted on SD or SLac media in the presence or absence of 1 mM Etn in ten-fold serial dilutions and were incubated for 2 or 7 days, respectively. (A) Disruption of ERMES component genes in psd1Δ leads to irreversible loss of mitochondrial function. (B) Disruption of EMC component genes does not affect the growth of psd1Δ. (C) Disruption of vCLAMP / HOPS complex genes aggravate the growth of psd1Δ. (D) Disruption of VPS13 does not affect the growth of psd1Δ. (TIF) [file pone.0215009.s010.tif]
