# Supplementary material for: Suppression of respiratory growth defect of mitochondrial phosphatidylserine decarboxylase deficient mutant by overproduction of Sfh1, a Sec14 homolog, in yeast
Source: PLoS One. 2019 Apr 8;14(4):e0215009. doi: 10.1371/journal.pone.0215009 (PMC6453485; doi:10.1371/journal.pone.0215009)
Supplement: S1 Table — (DOCX) [file pone.0215009.s011.docx]

**S1 Table. Primers used in this study.**

| Primer | Primer sequence (5’-3’) |
| --- | --- |
| PSD1-K1 | GCTCTAGCATTGGATCTTAC |
| PSD1-K2 | TACCACCTCCTTCGCAACTGG |
| delta-PSD1-A-r | TACGTTAATCACGATACGGCGCTGGCTTTGCTTTTCCTTC |
| delta-PSD1-B-f | CTGCTTAAGTCTGCGATTAAAAGCAATCATATGTAAAGTT |
| delta-PSD1-nest-f | AGCAAGAAATGTCCAAGATG |
| delta-PSD1-nest-r | TTGAAAGTTTGGGTATTCAA |
| ADE2-u | GCCGTATCGTGATTAACG |
| ADE2-l-2 | TTAATCGCAGACTTAAGC |
| PSD2-CU | TGCTCCTCCTCTGCGGTTAG |
| PSD2-CL | GATCCGATTGATATGGCACG |
| psd2-A-r | CGGCGGGGACAAGGCAAGCTCTTCGTCGTTGGATGCTCCT |
| psd2-B-f | ACGCCGCCATCCAGTGTCGAAGCCGCCGAAAAATTGGCTG |
| PSD2-delta-f | GATCTCTTTTTCTTGCTAAG |
| PSD2-delta-r | ATTATGCTAGATTGGGTTCCG |
| hph-fw | AGCTTGCCTTGTCCCCGCCG |
| hph-rv | TCGACACTGGATGGCGGCGT |
| ECT1-CU | GGAGCTGTTCTTAGACAC |
| ECT1-A-r | TTCTTTTTTCGACCGAATTCCAGTCAGTATATTTCAAG |
| ECT1-B-f | AATATACATCGCAGGGGGTTTCGAACGTAAATTAAATG |
| ECT1-CL | CAAACAAGTGGACCGAAC |
| ECT1-delta-f | CCTATTGGTGTTACTAGC |
| ECT1-delta-r | GCAAGGTTCCGGCAAGGG |
| TRP1-u | GAATTCGGTCGAAAAAAGAA |
| TRP1-l | AACCCCCTGCGATGTATATT |
| SFH1-del-f | TAAGTATGAATTTGCAAGGC |
| SFH1-del-r | AGGACAGGTTATTACACCAC |
| SFH1-CU | TATAAACCTTGAACCTGAGC |
| SFH1-CL | TCGATTGAGTGGGTTAGTGC |
| PSS1-del-f | GTGTGCAGACTTCTGTGCCAGACAC |
| LEU2-f | AACTGTGGGAATACTCAGGT |
| LEU2-r | AACGTTTCTTTCGCCTACGTGG |
| PSS1-LEU2-A-r | ACCTGAGTATTCCCACAGTTTTTTTAATATATAGTTTTAT |
| PSS1-LEU2-B-f | ACGTAGGCGAAAGAAACGTTTAGTTTTTGTTCATGCCTGG |
| PSS1-del-r | TCGGTGGTTTGTACATGGAG |
| PSD1-A-r(drug) | CGGCGGGGACAAGGCAAGCTGCTGGCTTTGCTTTTCCTTC |
| PSD1-B-f(drug) | ACGCCGCCATCCAGTGTCGAAAGCAATCATATGTAAAGTT |
| nat1-fw | AGCTTGCCTTGTCCCCGCCG |
| nat1-rv | TCGACACTGGATGGCGGCGT |
| MDM34-del-f | AACACGGCTAGCTAAGGGAA |
| MDM34-del-r | TTGTGGTCAGTCGTTTGATA |
| MDM34-CU | ACCAAACCCATACTAAAGTC |
| MDM34-CL | CACTATAAGATGCCCGTTAC |
| EMC1-CU | CCCCCAAGGCTCTCACTAAT |
| EMC1-del-A-r | AAACGTTGAATGAAGACAAATGCTATGGGGGAAGGGGAGG |
| EMC1-del-B-f | TTTTTTTCTCTTGAACTCGAATATTTACATACACATTATA |
| EMC1-CL | GGTCCTTTGAGAACCTGAAA |
| EMC1-del-A-f | TGCGGATTGCTTAGTTGTAC |
| EMC1-del-B-r | TCGAACGTCTTCTTCATATTAG |
| EMC2-CU | CCATGAGGTATAAACAACCG |
| EMC2-del-A-r | CGGCGGGGACAAGGCAAGCTTGTTTTATCTACTGCTTTCT |
| EMC2-del-B-f | ACGCCGCCATCCAGTGTCGAACGGAGCCAAAATTAGAAGA |
| EMC2-CL | GAATGTCTCCCTGTTACCCG |
| EMC2-del-A-f | CGCCCAGAGAACTTTAGCTA |
| EMC2-del-B-r | CAGAAATGTTTTGTGACAGC |
| EMC3-CU | GGCTCATATTCACCAGAAGA |
| EMC3-del-A-r | AAACGTTGAATGAAGACAAACTGCGGCGCCCTAGCTTGTT |
| EMC3-del-B-f | TTTTTTTCTCTTGAACTCGAGTTCGTGGCTATATATGCAC |
| EMC3-CL | ATCTTGCCGTTCTTGAGCAA |
| EMC3-del-A-f | TCTGCGAACTGTACATATCC |
| EMC3-del-B-r | GACTCTCTATATGCAATGGAGG |
| EMC5-del-f | ACGTCAGACTTATTGCTCTT |
| EMC5-del- r | GTTCCACTTACGCTAACATG |
| EMC5-CU | CAACAGCGACGAAATCACAC |
| EMC5-CL | AGGGCTTGTCTGTACCTCAG |
| EMC6-del-f | TGACATTGATTATTATATATGAA |
| EMC6-del-r | CGTTCTCCTTTCACTTTTTACT |
| EMC6-CU | GTGAGGGAATTCGATTCACC |
| EMC6-CL | AAAATGCGGAGGTGTGATTA |
| VPS39-del-f | GCTTGCTTTTTTAATTAGAC |
| VPS39-del-r | GCATTGCTCGTCATACGATA |
| VPS39-CU | CTCCTGCAAATTCATACTCT |
| VPS39-CL | AGAAAATCTTGAGTAATAGA |
| YPT7-del-f | CAGCTCCGTAACTCGAAAAG |
| YPT7-del-r | CACGTTTTGTTGAGTTATAT |
| YPT7-CU | GAGATAAAGAGTTTGAAAGA |
| YPT7-CL | ATTTTGTTAGGTAGTCATGT |
| VPS13-del-f | TGTTGGTTTTCAAGAGCTTGG |
| VPS13-del-r | TCAATCCCTTCCTTTAGCCT |
| VPS13-CU | GGTGGTAACTGATCAGTCCT |
| VPS13-CL | TCCTTCTCAATAGCACGTTC |
| SFH1-EcoRI-f | GGAATTCCTTAATTATACGTAAAACAT |
| SFH1-SalI-r | ACGCGTCGACATATGATGTTCGACTTTGGG |
| SFH2-EcoRI-f | GGAATTCACTCGTCATGTCACACGGAG |
| SFH2-SalI-r | ACGCGTCGACTGATTTTTCGTATAGCAGGC |
| SFH3-BamHI-f | CGCGGATCCTCTATGAGCCACGGAAATAG |
| SFH3-SalI-r | ACGCGTCGACGTTCGCCAGAGCTGCTAATG |
| SFH4-BamHI-f | CGCGGATCCCATGCCATTTAACCTGAATA |
| SFH4-SalI-r | ACGCGTCGACGAATCATTGACGGAGCCATT |
| SFH5-BamHI-f | CGCGGATCCAATCTATTATGAACAAGTGG |
| SFH5-SalI-r | ACGCGTCGACTCTTCTTGTTATCTTATCTC |
| SEC14-EcoRI-f | CCGGAATTCGATGCCGTACG TGTCGTCTG |
| SEC14-PstI-r | AAAACTGCAGCATATGTATT CTATAAGATA |
| SFH1-ST-A-r | TACCCTTCAAGTCCAGCACAATGCAAATAGTTTCAATAAGGTAGCCAG |
| SFH1-ST-B-f | CTGGCTACCTTATTGAAACTATTTGCAATGTGCTGGACTTGAAGGGTA |
| SFH1-R61A-f | TATTGCGATTTCTACGGGCAGCTAAATTTGATATCAATGCTAG |
| SFH1-R61A-r | CTAGCATTGATATCAAATTTAGCTGCCCGTAGAAATCGCAATA |
| SFH1-T238D-f | AACCCTTCTTGGATCCAGTAGATGTGTCAAAAATCTTTATTCT |
| SFH1-T238D-r | AGAATAAAGATTTTTGACACATCTACTGGATCCAAGAAGGGTT |
| SFH1-L179W-f | TTGAAACTTCGTGCACTGTGTGGGACTTGAAGGGTATCTCACT |
| SFH1-L179W-r | AGTGAGATACCCTTCAAGTCCCACACAGTGCACGAAGTTTCAA |
| SFH1-I196W-f | CTTATCATGTGCTTTCCTATTGGAAAGATGTTGCAGATATCAG |
| SFH1-I196W-r | CTGATATCTGCAACATCTTTCCAATAGGAAAGCACATGATAAG |
| SFH1-Y113C-f | CCAAAATGTACCCTCAGTATTGTCATCACGTTGATAAGGATGG |
| SFH1-Y113C-r | CCATCCTTATCAACGTGATGACAATACTGAGGGTACATTTTGG |
| SFH1ctag-SacI-r | CGAGCTCGCTGGTAACAGTAAA |
| 3xHA-SacI-f | CGAGCTCTACCCATACGATGTTCCTGA |
| 3xHA-SalI-r | ACGCGTCGACTCAGCACTGAGCAGCGTAATCT |
| EGFP-SacI-f | CGAGCTCGTGAGCAAGGGCGAGGAGCT |
| EGFP-SalI-r | ACGCGTCGACTTACCTTGTACAGCTCGTCCA |
| ZZ-SacI-f | CGAGCTCGCGCAACACGATGAAGCCGT |
| ZZ-SalI-r | ACGCGTCGACTTAGCTCGAATTCGCGTCTAC |
| His8-SFH1-fw | CATGCCATGGGTCATCATCATCATCATCATCATCATATGACAACCAGCATACTCGATAC |
| His8-SFH1-rv | CGAGCTCTTAGCTGGTAACAGTAAATTTACCAAAAATG |
| PSD1-BamHI-f | CGCGGATCCTGAGACAAGATGGTGGTACT |
| PSD1-KpnI-r | CGGGGTACCAACGGATTCAGAATCTTATA |
| PSD2-HindIII-f | CCCAAGCTTTCACGACTCAAAGAAAAAAA |
| PSD2-SalI-r | CCGTCGACGTACTTACTGCACTGTAAAT |
| PSD2ctag-SalI-r | CGGTCGACTAGCCCAGCAAAATCTTTAT |
| SalI-FLAG-f | ACGCGTCGACGACTACAAAGACCATGACGG |
| FLAG-GAPDHt-r | TTGGCAACGTGTTCAACCAACTTGTCATCGTCATCCTTGT |
| FLAG-GAPDHt-f | ACAAGGATGACGATGACAAGTTGGTTGAACACGTTGCCAA |
| GAPDHt-SpeI-r | CCACTAGTTCAATCAATGAATCGAAAAT |
| PstI-PSS1-f | AACTGCAGGATTATAGAGCTTATAGCTA |
| PSS1-SacI-r | CCGAGCTCTTCTTAATATACAAAGAATG |
| BamHI-PSS1-f | GCGGATCCGATTATAGAGCTTATAGCTAC |
| PSS1ctag-KpnI-r | GGGGTACCTGGCTTTGGAATTTTCAAG |
| KpnI-EGFP-f | CGGTACCGTGAGCAAGGGCGAGGAGCT |
| PstI-DPL1-f | AACTGCAGACATGAATGTGACGTTTCCT |
| DPL1-SacI-r | CCGAGCTCTTATTTGTAGAAGGATTGTT |
| SalI-MDM34-f | CCGTCGACTCAACAGCTTCGAAACACTG |
| HindIII-MDM34-r | GGGAAGCTTAAGGATGATATTGTTCTATT |

Restriction sites are indicated by underlines.
